# Enriched Conformational Sampling of DNA and Proteins with a Hybrid Hamiltonian Derived from the Protein Data Bank

**DOI:** 10.3390/ijms19113405

**Published:** 2018-10-30

**Authors:** Emanuel K. Peter, Jiří Černý

**Affiliations:** Institute of Biotechnology of the Czech Academy of Sciences, BIOCEV, Průmyslová 595, 252 50 Vestec, Czech Republic

**Keywords:** enhanced molecular dynamics simulations, protein folding, DNA simulation

## Abstract

In this article, we present a method for the enhanced molecular dynamics simulation of protein and DNA systems called potential of mean force (PMF)-enriched sampling. The method uses partitions derived from the potentials of mean force, which we determined from DNA and protein structures in the Protein Data Bank (PDB). We define a partition function from a set of PDB-derived PMFs, which efficiently compensates for the error introduced by the assumption of a homogeneous partition function from the PDB datasets. The bias based on the PDB-derived partitions is added in the form of a hybrid Hamiltonian using a renormalization method, which adds the PMF-enriched gradient to the system depending on a linear weighting factor and the underlying force field. We validated the method using simulations of dialanine, the folding of TrpCage, and the conformational sampling of the Dickerson–Drew DNA dodecamer. Our results show the potential for the PMF-enriched simulation technique to enrich the conformational space of biomolecules along their order parameters, while we also observe a considerable speed increase in the sampling by factors ranging from 13.1 to 82. The novel method can effectively be combined with enhanced sampling or coarse-graining methods to enrich conformational sampling with a partition derived from the PDB.

## 1. Introduction

Molecular dynamics (MD) simulations of biomolecular systems are important for an understanding of the molecular basis of biological processes [1], protein folding, and protein aggregation [2]. A wide range of applications of MD have emerged and successfully helped to predict efficient inhibitors used as drugs for various diseases [3]. Although the MD method is widely used, the efficiency of MD is limited by the underlying force field parameter set and the accessible timescales [4,5]. A larger number of force field parameter sets and efficient algorithms have been developed to describe DNA and proteins in solution [6,7,8,9,10,11,12,13,14,15,16], and a vast range of enhanced sampling and coarse-graining methodologies have emerged as efficient techniques for the determination of free energy partitions that depend on the conformational space [5,17,18,19,20,21,22,23,24,25,26,27,28,29,30,31,32,33,34,35,36,37,38,39,40,41,42,43,44,45,46,47,48,49,50,51,52,53,54,55,56,57,58,59,60,61,62,63]. Independent of the computational developments, an increasing number of experimental structures of proteins and DNA became publicly available from the Protein Data Bank (PDB [64]). The structural datasets demonstrate the structural variability of DNA and proteins, knowledge which has been necessary for understanding how DNA and proteins adopt their structures upon binding to ligands or other biomolecules in signaling processes. For example, it has been shown that DNA can adopt a large variety of structural conformers in addition to the canonical ’B’ form found by Watson and Crick [65], as defined in the structural alphabet of DNA [65,66,67,68]. The variety of possible structures ranges from double to triple and quadruple helices [69,70], DNA junctions, as well as parallel helices and hairpins [71,72]. An accurate parameterization which makes all conformers of DNA and proteins accessible in MD simulation with realistic statistical weights still remains a challenging task due to the large number of degrees of freedom even in a single dinucleotide building block (step) or the sequences of several protein secondary structure elements.

**Figure 1:**
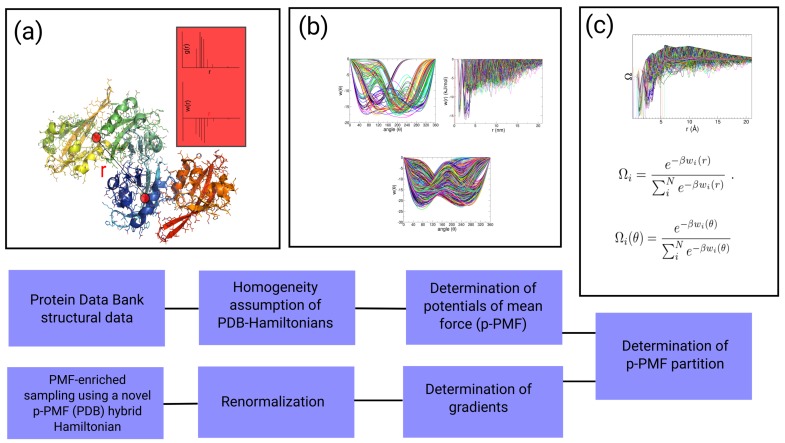
The potential of mean force (PMF)-enriched sampling technique involves a number of technical and theoretical steps for the application of *pseudo*-potentials of mean force (p-PMFs) in the enhanced sampling of proteins and DNA. (**a**) Scheme for the determination of pair correlations $g(r_{ij})$ and correlation functions in the torsion space $g(\theta_{k})$; (**b**) p-PMFs in the radial and the torsion space generated from the PDB data; (**c**) Partition function $\Omega (r_{ij})$ used in the propagation of the system. The gradient of the partition function along $r_{ij}$ is used for the propagation of the system. In the panel below, we show the different technical, algorithmic, and theoretical steps for the generation of the PDB-derived hybrid Hamiltonian for the enhanced sampling of proteins and DNA.

In this paper, we introduce an approach called potential of mean force (PMF)-enriched sampling, which uses partition functions for the conformational space as described in the Protein Data Bank (PDB). The technique enriches the conformational space of a DNA or protein molecule in the MD simulation with structural partitions from the PDB and accelerates transitions through the definition of a hybrid Hamiltonian consisting of the underlying force field and a Hamiltonian derived from the PDB partition (see Figure 1). For this approach, we derived effective partitions for *pseudo*-potentials of mean force (p-PMF) from the PDB. The PDB structures resemble a large number of different Hamiltonians with different salt-concentrations, volumes, pressures, and temperatures, which makes the direct determination of potentials of mean force difficult. We solved that problem through the introduction of an approximation of a *quasi*-homogeneity within the collected data and the definition of an error estimate that we introduce during the approximation procedure. The partition resembles a PDB-related probability density with an associated error and an associated energy, from which we can define a gradient of the auxiliary Hamiltonian used as a bias in MD simulations. The simulation approach can significantly shift the partitions from the averages defined by the force field toward the PDB partition, which, to the best of our knowledge, does not exist at present. We measured the radial distribution functions *g(r)* for more than 1800 non-redundant X-ray structures of DNA and 24,300 protein structures. We generated effective *pseudo*-potentials of mean force (p-PMF) using the pair correlation functions and built individual p-PMF topologies to define an auxiliary Hamiltonian based on a p-PMF partition, which adaptively changes throughout the MD integration. We validated our method using simulations of dialanine, the folding of TrpCage [73], and the Dickerson–Drew DNA dodecamer (PDB: 1bna) [74], on which we tested different force fields—AMBER99, AMBER12sb, AMBER14sb—and compared our results with MD simulations of the same molecule. In the analysis of the DNA data, we assigned the resulting trajectories to the structural alphabet of DNA and found that the PMF-based method accelerates the sampling of DNA by a factor of up to 20.0 compared to standard MD simulations of the same DNA molecule. In the sampling of the dialanine peptide and the folding of TrpCage, we observed effective acceleration factors of 13.1 and 82.3. In each of the examples, we observed the validity of the method, which improves the sampling of conformer partitions toward the statistics represented in the PDB. As a significant advantage, the PMF-enriched sampling can be coupled to other enhanced sampling techniques or coarse-grained simulation methods to sample conformations within the partition described by the PDB.

## 2. Methods

Force field parameters describe the interactions in the system, which we use for propagation in the MD simulation. Conventionally, the parameter sets are improved in their accuracy and validated by a comparison with available data. In this article, we propose an approach called ’PMF-enriched’ sampling, which increases the accessible conformational space of the underlying force field parameter sets. For our approach, we combined data available from the Protein Data Bank (PDB) with the Hamiltonian described by the force field data set. In order to achieve that goal, we defined an auxiliary Hamiltonian H(B) from the PDB, which we combined with the Hamiltonian H(A) defined in the force field. Therefore, we state that the structures deposited in the PDB resemble a wide range of possible configurations which proteins and DNA can adopt. Potentials of mean force (PMF) extracted from the PDB have been effectively used in the development of coarse-grained interactions [75]. The PMFs represent the mean pair interaction energies, as described in the radial pair correlation function of a pair of atoms, within a statistical average [76]. That relation has also been used for the parameterization of protein coarse-grained models [77,78,79] and PMF data has been used as a measure for the investigation of average protein conformations [80,81].

For our approach, called *PMF-enriched* sampling, we consider that the structural partition, in principle, can serve as a source of parameters describing the sequence-specific patterns of interactions and the flexibility of sequence-specific patterns. However, the experimental conditions for the determination of protein or DNA structures, as well as thermodynamic quantities, such as the number of atoms, the volume of the crystal unit cell, and/or the pressures, differ between each structure deposited in the PDB, which makes the direct exploitation of the PDB data difficult, since a direct Boltzmann distribution cannot be determined. In order to solve the problems associated with the direct potentials of mean force, we formulated an approximation of a *quasi*-homogeneity as a property of the PDB data that we apply through the introduction of a homogeneous atom-density to the potentials of mean force (which we call *pseudo*-potentials of mean force (p-PMF)). As a consequence, we introduce an intrinsic error into each single p-PMF, which leads to a systematic error if we propagate the system with that set of p-PMFs. To tackle the problem of the systematic error, we approximate the introduced error to be constant over the whole distance and define a system-specific partition function for the p-PMFs, leading to the definition of an energy quantity. While the energy is associated with the systematic error, the constant error is canceled when we define the gradient of the p-PMF-related partition function defining the auxiliary Hamiltonian H(B) to correct the original partition, as given in the force field H(A). Finally, we combine the two Hamiltonians using a renormalization that is dependent on a coupling factor α to obtain a new effective Hamiltonian H(C) containing the PDB-related correction H(B).

We compared the PMF-enriched TrpCage simulation results with a simulation using an extension of the enhanced *path-sampling* technique, a method which defines a path variable *L*, which is determined *on the fly* and does not require a priori knowledge of the reaction coordinates [82]. That path-variable dL is derived from the intrinsic dynamics of the system and accelerates the sampling along the pathways of action. Through that procedure, the enhanced sampling methodology accelerates the sampling of a system, while a minimal set of input parameters and no a priori information of reaction pathways or product states is required. At the same time, the principal conservation law of the reduced action is used to control the ergodicity of the dynamics. We extended the method to the sampling along multiple pathways $dL_{ik}$ and a renormalization of the bias to the underlying unbiased Hamiltonian [83].

### 2.1. Theory

We start considering an ideal case of a structural databank of proteins and DNA, in which each structural member is determined with an extremely high resolution and identical experimental conditions (identical volumes, identical temperatures, and pressures), and every potential conformation of each existing protein sequence is represented by a member in the set of data. In this ideal case, the dataset can be used for the determination of a single Hamiltonian *H* to describe the dynamics of proteins and DNA. Each angular g(θ) and radial distribution g(r) (categorized into bonded and non-bonded distributions) and correlation functions of higher order could ideally be used through the determination of corresponding potentials of mean force w(θ) and w(r). In the NVT-ensemble, such dataset could be described by one single partition function:

(1) $$Z=\int_{-\infty }^{\infty }\ldots \int_{-\infty }^{\infty }\int_{0}^{2\pi }\ldots \int_{0}^{2\pi }e^{-\beta (w\left(r^{N}\right)+w\left(\theta^{N}\right))}dr^{1}\ldots dr^{N}d\theta^{1}\ldots d\theta^{N}\;,$$

where $w_{N}$ stands for the potential energy function, *r* stands for the coordinate, and *N* is the total number of particles. However, the resolution of X-ray structures in the PDB often lies between 1 and 3.5 Å, and the experimental conditions vary between each structure in the databank. In a very general sense, we express the consequence from the intrinsic differences between the PDB entries as a general quantity δw, by which each structure deviates from the idealized partition. We generally find three potential errors, which have to be solved if a PDB dataset will be used as a potential to enrich the sampling in a MD simulation. (1) The difference in the experimental conditions can lead to strong shifts in the partitions of conformations. That error is approximately constant if the selection of PDB structures for the generation of p-PMFs leads to an approximately identical energy landscape as the underlying landscape of the simulated system, i.e., the selection of PDB structures for the generation of p-PMFs has to be necessarily system-dependent. If that error is approximately constant, the term δw vanishes due to the application of the partition Ω, as described later. (2) A second potential error also arises from the selection of PDB structures and can lead to deviations in the sampling of secondary structures if the p-PMFs contain minima arising from conformations in the PDB that do not belong to the conformation space of the system of interest (see section Results: Simulations of TrpCage). (3) The conformation space in the PDB datasets is limited by the number of structures available, which leads to undefined regions between minima within the potential *w*. At the same time, all PDB structures resemble compact and folded structures. Thus, the PDB-derived potential has to be necessarily combined with a standard force field using a renormalization scheme of the Hamiltonian, which leads to a new hybrid Hamiltonian. The resulting dynamics based on that Hamiltonian is enriched by configurations from the PDB, but the trajectory is limited in the conformation space by the quality and the structures in the datasets used for the determination of the p-PMFs.

### 2.2. Associated Error δw

Through the selection of non-redundant structures of proteins with a resolution higher than 2.5 Å (over 24,300 X-ray structures, where the sequence length is larger than 50 residues, no DNA or RNA is present in the system, and the set of structures contains less than 70% sequence identity) and DNA (over 1800 X-ray structures, with a crystallographic resolution higher than 3.0 Å, containing DNA longer than 5 nucleotides, no RNA is present, and the structures are allowed to contain proteins longer than 20 amino acids), we approximate that the introduced error arising from the experimental conditions,

(2) δw≈const.,

is approximately constant within a limited radius of r=2.5 nm, in which we determined the *pseudo*-potentials of mean force (p-PMF). However, another effect from the averaging over PDB data can lead to a smaller additional error. The PDB data contains various secondary structural forms, which can lead to deviations along the folding pathway if particular elements of the biomolecule (especially proteins) only contain one particular secondary structure along their folding pathway. That means that a proportion of minima in the p-PMFs arising from other secondary structural elements can guide the system toward partially artificial conformations. The associated error δw can be minimized using a guided and system-dependent selection of PDB structures with an approximately similar number of atoms and secondary structural content in the beginning of the simulation, in contrast to averaging over a broad dataset, as performed in the study described in this article. As shown in the PMF-enriched simulations of TrpCage, the guided selection of PDB data for the p-PMFs can improve the convergence to helical conformations, while the associated error in the simulation of the double-helical DNA (as shown in the PMF-enriched simulations of the Dickerson–Drew DNA dodecamer) is significantly lower, since the PDB structures employed for the p-PMF determination are mostly double helical. For simulations of both DNA and proteins, we propose to complement the data from simulations with a number of selected PDB structures to minimize the error and to reach a set of p-PMFs for which deviations, which arise from secondary structures and experimental conditions, are approximately equal or close to a value of 0. In this study, we approximated the error δw to be constant due to the selection criteria and applied the complete PDB dataset to the folding simulations of TrpCage and the Dickerson–Drew DNA dodecamer. An alternative procedure is the generation of the p-PMF data in enhanced sampling or long-time MD simulations of the same system, which minimizes all errors but, at the same time, would be dependent on the applied force field and not on the experimental PDB data. That procedure finds its analogy in the iterative Boltzmann inversion procedure or the force-matching method to define potentials in coarse-grained MD [84,85].

### 2.3. Renormalization of the Hamiltonian

To cope with the intrinsic difference δw between an idealized case and the real underlying partition due to the incompleteness of the data, we used a standard force field parameter set as a reference and also added the nonideal PDB-derived data to that Hamiltonian through renormalization. Therefore, we consider that the interactions in any system are described by a Hamiltonian H(A) based on the standard force field, which describes the effective forces used for its propagation with the time-step dt. Any property *X* as a result of sampling using the Hamiltonian H(A) leads to a corresponding probability *P* to match a certain value:

(3) P(X)=f(H(A)).

We defer to a large number of publications related to the validity or the invalidity of parameter sets describing the Hamiltonian of a protein H(A) and, especially, DNA systems, where the target property P(X) varies with the applied datasets [86,87,88,89,90,91]. We now turn to enhanced sampling methods, where an additional bias H(B) in the energy space changes the resulting probability $P^{\prime }$ to match a defined target property *X* to:

(4) $$P^{\prime }\left(X\right)=f\left(H\left(A\right)+H\left(B\right)\right)\;.$$

It is widely used to apply the additional bias H(B) along the collective variables describing the slowest degrees of freedom of the system, as, for example, in umbrella sampling [92] and related methods, such as Metadynamics [17], conformational flooding [93], or local elevation [28]. If the slowest degrees of freedom are not assigned correctly to the additional Hamiltonian H(B), the probability $P^{\prime }\left(X\right)$ is strongly affected and matches the property *X* in the wrong way.

We recently introduced a renormalization scheme to solve a problem that arises with the unbiased probability P(X), i.e., it can be strongly affected by the added bias H(B) [83]. In this scheme, added bias tending to accelerate the system is renormalized to the unbiased Hamiltonian H(A) in a way such that the added bias only equals a fraction of the unbiased Hamiltonian H(A) that is dependent on a coupling factor α. At the same time, we introduce a renormalization of the unbiased Hamiltonian by the same factor, leading to a new Hamiltonian H(C):

(5) $$H\left(C\right)=\frac{H(A)}{1+\alpha }+\frac{\alpha |H(A)|}{\left|H(B)\right|}H\left(B\right)\;.$$

In principle, the total then energy remains mostly unaffected, while another property has been introduced through the bias H(B). In other words, we generated a new probability distribution $P^{\prime \prime }\left(X\right)$ as a result of adding a particular bias H(B) that is dependent on a linear coupling factor α:

(6) $$P^{\prime \prime }\left(X\right)=f\left(\frac{H(A)}{1+\alpha }+\frac{\alpha |H(A)|}{\left|H(B)\right|}H\left(B\right)\right)\;.$$

Through this formalism, we can introduce properties to the original Hamiltonian without affecting the total energy of the system. In this work, we generated a set of auxiliary Hamiltonian from an set of *pseudo*-potentials of mean force (*p*-PMF) from radial pair distribution functions g(r) averaged over the protein data bank (PDB). In other words, the auxiliary Hamiltonian H(B) is added to the system in the form of a renormalized fluctuation; this is in contrast to a strong biasing in the energy space as is conventionally performed in umbrella sampling-related methods [17]. While the orientation of H(B) is explicitly derived from the definition given in the p-PMF (interatomic distance vectors), the bias acts on the system through a renormalized fluctuation. That confers an advantage: the propagation of the system remains ergodic as long as the coupling factor α is within a low range of approximately <$10^{-8}$. Considering the fact that bonded interactions in the biomolecular force field can contribute with gradients $>10^{4}-10^{5}$ kJ/mol/nm, coupling with a factor with a magnitude of $\alpha =10^{-8}$ corresponds to the order of magnitude that is typical of non-bonded interactions. As we mention later, the parameter α is coupled to a fluctuation parameter ϵ through a process coupled to a random number that can enhance the sampling of the system, while we obtain the correct time averages in the coupling equal to a value of α [83].

### 2.4. Auxiliary *p*-PMF from the PDB

Here, we first state that the applied PMF data from the PDB does not resemble a unique ensemble, since the Hamiltonian changes in approximately every single X-ray or NMR model deposited in the PDB, which is obvious if we consider the different number of atoms, the different volumes in the crystal unit cell, and the different thermodynamic conditions. In contrast to an energetical point of view, we start with a general statistical argument: that the PDB, in general, resembles a statistical dataset of conformations that a biomolecule can adopt, depending on its amino acid or DNA sequence. We determined the radial pair distribution functions $g(r_{ij})$ of a pair of atoms with index *i* and *j*, as well as distributions $g(\theta_{k})$ along torsion angles θ with index *k*, of a large set of different configurations and conformations, while we assumed a unique number of particles in each system (i.e., the same number of atoms *N* and the same volume *V*), using an identical factor $\rho =\frac{V}{N}$ to normalize the histograms over distances and volume increments $\frac{4}{3}\delta r^{3}\pi$:

(7) $$g\left(r_{ij}\right)=\frac{1}{\rho }\frac{\sum_{i\neq j}\delta \left(r_{i}-r_{j}\right)}{\frac{4}{3}\delta r^{3}\pi }\;,$$

over *N* pairs of atoms *i* and *j*. For the torsional space, we generate an analogous set of the angular partition function $g(\theta_{k})$:

(8) $$g\left(\theta_{k}\right)=\langle \delta \left(\theta -\theta_{k}\right)\rangle \;.$$

For the generation of histograms, we used the torsions Φ and Ψ for proteins and eight torsions (α, β, γδ, ϵ, χ, ζ) for DNA. From this normalized dataset over a statistical partition of torsional and radial distributions for various PDB structures, we generate a *pseudo*-potential of mean force (p-PMF), thereby neglecting the given fact that the real partition—in other words, a Boltzmann partition function—cannot be generated. We apply:

(9) $$w\left(r_{ij}\right)=-k_{B}T\ln\left(g\left(r_{ij}\right)\right)\;,$$

and

(10) $$w\left(\theta_{k}\right)=-k_{B}T\ln\left(g\left(\theta_{k}\right)\right)\;,$$

over $N_{k}$ torsions.

### 2.5. Statistical Partition of p-PMF for the Generation of Auxiliary Hamiltonian: PMF-Enriched Sampling

The individual statistical distributions from the PDB are normalized with respect to the other distribution functions in the system for the generation of a partition function based on the p-PMFs. Each p-PMF added to the system in the form of a bias is associated with an error related to the real Boltzmann distribution. In the following, we derive the formalism for the PMF-enriched sampling with the radial distribution function $g(r_{ij})$, while the same formalism is valid for the torsional distribution. We note that the associated error $\delta w(r_{ij})$ of each function $w(r_{ij})$ is individual with respect to the new resulting partition using a normalized density for the function $g(r_{ij})$. The function is written as:

(11) $$w\left(r_{ij}\right)=w\left(r_{ij}\right)+\delta w\left(r_{ij}\right)\;.$$

If we use this definition, we would additionally introduce an error based on the approximation of the uniformity of the PDB data (a uniform number of particles and a uniform volume). However, we can define a probability density $\Omega (r_{ij})$ as a function of the individual functions $w(r_{ij})$ within the same system, expressed as (using $\beta =\frac{1}{k_{B}T}$):

(12) $$\Omega \left(r_{ij}\right)=\frac{e^{-\beta w(r_{ij})}}{\sum_{i}^{N}e^{-\beta w(r_{ij})}}\;,$$

which also can be written as:

(13) $$\Omega \left(r_{ij}\right)=\frac{e^{-\beta w\left(r_{ij}\right)-\delta w\left(r_{ij}\right)}}{\sum_{i}^{N}e^{-\beta w\left(r_{ij}\right)-\delta w\left(r_{ij}\right)}}\;.$$

This expression can be reformulated into:

(14) $$\Omega \left(r_{ij}\right)=\frac{e^{-\beta w(r_{ij})}e^{-\beta \delta w(r_{ij})}}{\sum_{i}^{N}e^{-\beta w(r_{ij})}e^{-\beta \delta w(r_{ij})}}\;.$$

According to this definition, the probability density for all atoms described by a PMF fulfills the norm:

(15) $$\sum_{i}^{N}\Omega \left(r_{ij}\right)=1\;,$$

independent of the associated error, which we introduced by the approximation of a homogeneity in the PDB data. Through the selection of PDB structures that depend on the sequence redundancy and on the resolution being higher than 2.5 Å (proteins) and 3.0 Å (DNA) (see subsection Theory), which can be improved by the guided and system-dependent selection of PDB structures, we approximate that the associated error $\delta w(r_{ij})$ is approximately constant, and any coordinate of the system can be found with a probability density which depends on $\Omega (r_{ij})$. To describe a gradient as a bias, we define the change in the probability density along the measured distance and torsion coordinates for each atom *i* for which a p-PMF has been defined, while the energy of the bias depends on the coupling factor α and the magnitude of the unbiased gradient. In an ideal case, a quasi-Boltzmann-weighted free energy can be defined using the partition $\Omega (r_{ij})$:

(16) $$\Delta F\left(r_{ij}\right)=-k_{B}T\ln\Omega \left(r_{ij}\right)\;,$$

which is correlated with a comparatively strong error based on the approximation of a *quasi*-homogeneity in the PDB data. However, we can define a gradient of this energy, given as:

(17) $$\frac{d}{dr_{ij}}\Delta F\left(r_{ij}\right)=-k_{B}T\frac{1}{\Omega (r_{ij})}\frac{d}{dr_{ij}}\Omega \left(r_{ij}\right)\;.$$

Since we renormalize the added Hamiltonian H(B) by the linear factor α, we only use the vector component bias added to the system:

(18) $$\frac{d}{dr_{ij}}H_{i}\left(B\right)=\frac{d}{dr_{ij}}\Omega \left(r_{ij}\right)\;.$$

This is an advantage since the associated error within the probability density is approximately constant, and we obtain an approximately correct bias based on a uniformity approximation, which we introduced with the measurement of p-PMFs from PDB data. In other words, introducing the probability density and using the gradient of the probability density $\Omega (r_{ij})$ lead to a significant reduction in the error, since the error $\delta w(r_{ij})$ is approximately constant. We mention that the partition $\Omega (r_{ij})$ is re-evaluated every time step since every conformation corresponds to another value in each individual p-PMF $w(r_{ij})$ and $w(\theta_{k})$. The invariance of the change in the partition $\delta \Omega (r_{ij})$, according to the error $\delta w(r_{ij})$ along the coordinates $r_{ij}$ and $\theta_{k}$, is written as:

(19) $$\frac{d}{dr_{ij}}\delta \Omega \left(r_{ij}\right)\approx 0\;,$$

and

(20) $$\frac{d}{d\theta_{k}}\delta \Omega \left(\theta_{k}\right)\approx 0\;,$$

so that we obtain a gradient based on the partition in which the associated error introduced to the p-PMFs is compensated. Finally, we express the gradient added as bias $\frac{d}{dr_{ij}}H\left(B\right)$ to the system after the renormalization:

(21) $$\begin{array}{ccc} \frac{d\Omega (r_{ij})}{dr_{ij}} & = & (-\beta \frac{dw(r_{ij})}{dr_{ij}}e^{-\beta w(r_{ij})}\sum_{i}^{N}e^{-\beta w(r_{ij})}\\ & - & e^{-\beta w(r_{ij})}\sum_{i}^{N}\beta \frac{dw(r_{ij})}{dr}e^{-\beta w(r_{ij})})\\ & \times & {\left(\sum_{i}^{N}e^{-\beta w(r_{ij})}\right)}^{-2}\;, \end{array}$$

and

(22) $$\begin{array}{ccc} \frac{d\Omega (\theta_{k})}{d\theta_{k}} & = & (-\beta \frac{dw(\theta_{k})}{d\theta_{k}}e^{-\beta w(\theta_{k})}\sum_{k}^{N_{k}}e^{-\beta w(\theta_{k})}\\ & - & e^{-\beta w(\theta_{k})}\sum_{i}^{N}\beta \frac{dw(\theta_{k})}{d\theta_{k}}e^{-\beta w(\theta_{k})})\\ & \times & {\left(\sum_{k}^{N_{k}}e^{-\beta w(\theta_{k})}\right)}^{-2}\;. \end{array}$$

### 2.6. Propagator

Using the expressions (5) and (18), we combine the gradient along the probability density with the Hamiltonian described by the underlying force field parameter set to obtain a modified Hamiltonian H(C), in which the Hamiltonian described by the force field and that described by the PDB partition are combined in a manner that is dependent on a linear coupling factor α:

(23) $$\begin{array}{c} \nabla \left(H\left(C\right)\right)=\frac{1}{1+\alpha }\nabla \left(H\left(A\right)\right)+\alpha \frac{\left|\nabla H(A)\right|}{\left|\nabla H(B)\right|}\nabla H\left(B\right)=\\ =\frac{1}{1+\alpha }\nabla U+\alpha (\frac{\left|\nabla U\right|}{|\frac{d\Omega (r_{ij})}{dr_{ij}}|}\frac{d\Omega (r_{ij})}{dr_{ij}}+\frac{\left|\nabla U\right|}{|\frac{d\Omega (\theta_{k})}{d\theta_{k}}|}\frac{d\Omega (\theta_{k})}{d\theta_{k}})\;, \end{array}$$

where *U* stands for the potential energy described by the applied force field.

### 2.7. Shift in the Free Energy Partition

In a very general way, the propagation along Equation (23) guides the system toward a changed partition depending on the linear coupling factor α. In the case of the canonical ensemble, the expectation value of a quantity *X* is given by:

(24) $$\begin{array}{ccc} <X> & = & \frac{Xe^{-\beta H(C)}}{\int e^{-\beta H(C)}drdp}=\\ & = & \frac{Xe^{-\beta \left(\frac{H(A)}{1+\alpha }+\frac{\alpha |H(A)|}{\left|H(B)\right|}H\left(B\right)\right)}}{\int e^{-\beta \left(\frac{H(A)}{1+\alpha }+\frac{\alpha |H(A)|}{\left|H(B)\right|}H\left(B\right)\right)}drdp}\;. \end{array}$$

In general, the new expectation value <X> is expressed as:

(25) $$<X>=\frac{<X(A)>}{1+\alpha }+\alpha <X\left(B\right)>\;,$$

which we reach through the addition of the second Hamiltonian H(B), which is expressed by the p-PMF partition Ω derived from the PDB, to the system. As expressed in Equation (25), the original property obtained using the Hamiltonian H(A) is complemented with another property from the Hamiltonian H(B) that depends on a coupling factor α. As shown in the Results section, our formalism successfully shifts the partition from the averages described by the force field toward a statistical partition derived from the PDB. We tested the validity of our approach using folding simulations of TrpCage and the conformations of the Dickerson–Drew DNA dodecamer, also described in the Results section.

### 2.8. Coupling Parameters α and ϵ

In order to facilitate transitions in the system, we allowed fluctuations of the α parameter, where α(t) at time *t* follows a process dependent on a uniform random number ξ∈[0,1] and the fluctuation parameter ϵ, defined by the equation:

(26) α(t)=α(1−ξ)×ϵ,

leading to the average value:

(27) 〈α(t)〉=α,

over a longer simulation period, while ϵ describes the relative width of the fluctuation of the forces around the average value of the coupling factor α. Thus, we obtain the same time average, while we accelerate the sampling through the inclusion of fluctuations in the system [83]. In the simulations, we varied the α parameter in a range from $10^{-9}$ (DNA) to $10^{-5}$ (dialanine). The magnitude of α and ϵ depends on the transitions, which should be sampled in the system, as well as the energy needed to surmount the barriers. In the case of DNA, larger coupling parameters $\alpha >10^{-8}$ lead to strong fluctuations within each dinucleotide step so that *syn*-conformations, and even non-helical or unpaired DNA conformations, are sampled. Higher coupling values in the simulations of TrpCage leads to population maxima in the unfolded region due to stronger dihedral transitions. In other words, the applied Hamiltonian H(B) defined by a broad PDB-averaging in the torsional and the radial space needs to be coupled to a lesser extent in simulations of the folded state than for a less complex system, like dialanine, where larger coupling parameters can be used also. A guided system-specific selection of datasets for the generation of p-PMF data can allow a uniform set of coupling values independent of the systems of interest. Additionally, we mention the effect of the height of the absolute values of the gradients, which is larger for DNA than for a system of the size of dialanine. The different absolute magnitudes of the unbiased gradients in the different systems also justify the use of coupling parameters with a varying magnitude.

### 2.9. Algorithm

- Read p-PMF data $w(r_{ij})$ and $w(\theta_{k})$ for relevant pairs of atoms and sequence.
- Start loop over MD-steps.
  - Measure coordinates $r_{ij}\left(t\right)$ and $\theta_{k}\left(t\right)$.
  - Determine values $w(r_{ij}\left(t\right))$, $w(\theta_{k}\left(t\right))$.
  - Determine partitions $\Omega (r_{ij}\left(t\right))$ and $\Omega (\theta_{k}\left(t\right))$ for $r_{ij}\left(t\right)$ and $\theta_{k}\left(t\right)$.
  - Determine gradients.
  - Add bias after renormalization to the unbiased gradient.

### 2.10. Path-Sampling Method

We validated our results of the PMF-enriched simulations of TrpCage using simulations that applied an extension of the recently published *path-sampling* method [82]. The *path-sampling* method defines the bias *s* used for the accelerated sampling along multiple path increments $dL_{ik}$ (for pathways *i* and atom indices *k*), as well as the modification of the bias to its principal components, so it is adaptively changed into the bias $s^{\prime }$ that is dependent on a distance restraint *r*, as obtained by experimental data [82]. For the derivation of the method, we consider that the simulated system in an equilibrium simulation propagates along a pathway with the general condition that the reduced action *L*, as a function of momentum *p* and positions *q*, remains constant [94,95]:

(28) L=∮pdq=η=const..

A local change in *L* at any time *t* within a time interval dt is defined by:

(29) $$\frac{dL(t)}{dt}=\frac{d}{dt}\left(pdq\right)=\frac{dp}{dt}dq+p\frac{dq}{dt}\;.$$

We obtain the following differential at time *t*:

(30) dL(t)=pdq+dpdq=(p+dp)dq.

In our recent work, the exploration of the pathway of action depended on the coupling time intervals $\tau_{1}$ (adaptive bias MD) and $\tau_{2}$ (*path-sampling*) in which the gradient has been evaluated [82]. We extended the formalism to sampling within $N_{R}$ multiple biases (and optional $N_{S}$ multiple simulations). We redefined Expression (28) to apply to a multiple sampling in multiple bias paths along $N_{R}$ multiple biases, which the system can undergo simultaneously:

(31) $$\begin{array}{ccc} L_{s} & = & \oint pdq+\sum_{i}^{N_{R}}\sum_{k}^{N}(dL_{i,k}\\ & + & dL_{\sigma_{r,i,k}}\left(dL_{i,k}\right))=\eta =\;const\;, \end{array}$$

where the two path increments $dL_{i,k}$ and $dL_{\sigma_{r,i,k}}\left(dL_{ik}\right)$ are derived from the adaptive bias MD and the *path-sampling* components of the hybrid algorithm [82]. Both components are added to the unbiased path ∮pdq in a way that the principle of conservation of the action integral is obeyed. In other words, the action integral has to remain constant upon the addition of the bias in order to sample the system along an equilibrium trajectory. Taking into account that protein systems especially, and aqueous solutions generally, behave heterogeneously in relation to their relaxation behavior (depending on their actual state) which extends to multiple biases, coupling is performed with a finite set of different relaxation times, $\tau_{1_{ik}}$ and $\tau_{2_{ik}}$, for $N_{R}$ biases with index *i* and *k* individual atom indices, leading to a more efficient sampling of dynamically heterogeneous systems. That way, we apply that individual number of $N_{R}$ biases within each individual bias *i*, for which a bias *s* is re-evaluated within periods $\tau_{1_{ik}}$ and $\tau_{2_{ik}}$ for the atom with index *k* and is applied to the same period. We define the individual time periods $\tau_{1_{ik}}$ and $\tau_{2_{ik}}$ over $N_{R}$ multiple pathways with the atom index *k* as:

(32) $$\tau_{1_{i=N_{R},k}}=\sum_{i=1}^{N_{R}}\tau_{1_{k_{i}}}\;,$$

and

(33) $$\tau_{2_{i=N_{R},k}}=\sum_{i=1}^{N_{R}}\tau_{2_{k_{i}}}\;.$$

Although we apply constant values $\tau_{1}$ and $\tau_{2}$ to all atoms, we introduce the notation with an additional index *k*, which would mean that each individual atom *k* can potentially be coupled to an individual value $\tau_{1_{ik}}$ or $\tau_{2_{ik}}$, which might lead to a higher accuracy for capturing the individual relaxation times of each atom in the system. For example, a system coupled to $N_{R}=5$, $\tau_{1}=$ 1 ps, $\tau_{2}=$ 2.5 ps is sampled with path-dependent biases coupled to the times $\tau_{1_{ik}}=\left\{1,2,3,4,5\right\}$ ps and $\tau_{2_{ik}}=\left\{2.5,5,7.5,10.0,12.5\right\}$ ps. We used characteristic time periods ranging from 10 ps to 1 ns. The two forms of the bias *s* depend on two independent coupling times, $\tau_{1_{ik}}$ and $\tau_{2_{ik}}$ ($\tau_{1}$ and $\tau_{2}$ correspond to $\tau_{1}$ for adaptive bias MD and the period $\tau_{2}$ for the collection of *path-sampling* coordinates). In particular, the two separate increments $dL_{\sigma_{r,i,k}}\left(dL_{i,k}\right)$ and $dL_{i,k}$ are evaluated within two separate modules called adaptive bias MD and *path-sampling*, which we called the hybrid *path-sampling* algorithm.

For the *adaptive bias MD* section of the algorithm, we derived a history-dependent bias *s* of the form:

(34) $$\begin{array}{c} s=\sum_{i}^{N_{R}}\sum_{k}^{N}\eta_{ik}^{\prime }\left(t\right)dL_{ik}=\sum_{i}^{N_{R}}\sum_{k}^{N}\eta_{ik}^{\prime }\left(t\right)\left(p_{k}+dp_{k}\right)dq_{k}\;. \end{array}$$

using a number of $N_{R}$ biases in which the bias is re-evaluated within periods of $\tau_{1_{ik}}$ for the bias with index *i* and atom *k*. As we introduced in our previous work, we define the corresponding force $F_{b}\left(t\right)$ at time *t* and use the time derivative of *s*: $\frac{d}{dt}s=\dot{s}$:

(35) $$\begin{array}{ccc} F_{b}\left(t\right) & = & \dot{s}\\ & = & \sum_{i}^{N_{R}}\sum_{k}^{N}[\eta_{ik}^{\prime }\left(t\right)\frac{d}{dt}\left[\left(p_{k}+dp_{k}\right)dq_{k}\right]\\ & + & \dot{\eta_{ik}^{\prime }}\left(t\right)\left(p_{k}+dp_{k}\right)dq_{k}]\;. \end{array}$$

As we defined in Equation (28), the added bias has to fulfill the condition $\lim_{t\to \infty }<dL{>}_{t}\approx 0$ in order to sample the system at equilibrium. That also implies that the averages of $\eta_{ik}$ have to fulfill $<\eta_{ik}^{\prime }\left(t\right)>=0$ and $\langle \frac{d\eta_{ik}^{\prime }}{dt}\rangle \approx 0$. To enhance sampling along a history-dependent pathway in adaptive bias MD, we employed a coarsening, expressed by:

(36) $$\begin{array}{ccc} \frac{d}{d\tau_{1_{ik}}}\dot{s} & = & \sum_{i}^{N_{R}}\sum_{k}^{N}\frac{d}{d\tau_{1_{ik}}}\times ((\eta_{ik}^{\prime }\left(t\right)\frac{d}{dt}\left[\left(p_{k}+dp_{k}\right)dq_{k}\right]\\ & + & \dot{\eta_{ik}^{\prime }}\left(t\right)\left(p_{k}+dp_{k}\right)dq_{k})). \end{array}$$

By taking into account that $\frac{d}{d\tau_{1_{ik}}}\left(\dot{\eta_{ik}^{\prime }}\left(t\right)\left(p_{k}+dp_{k}\right)dq_{k})\right)\approx 0$, we use this formalism to define the differential over finite time increments $\tau_{1_{ik}}$ to coarse-grain the dynamics and to increase the computational efficiency, which leads to an expression for the corresponding force in adaptive bias MD:

(37) $$\begin{array}{ccc} F_{b}\left(\tau_{1}\right) & = & \frac{d\dot{s}}{d\tau_{1}}d\tau_{1}\\ & = & \sum_{i}^{N_{R}}\sum_{k}^{N}[\eta_{ik}^{\prime }\left(t\right)\frac{d}{d\tau_{1_{ik}}}\left(\frac{d}{dt}\left[\left(p_{k}+dp_{k}\right)dq_{k}\right]\right)d\tau_{1_{ik}}\\ & + & \frac{d\eta_{1_{ik}}^{\prime }}{d\tau_{1_{ik}}}d\tau_{1_{ik}}\left(\frac{d}{dt}\left[\left(p_{k}+dp_{k}\right)dq_{k}\right]\right)]\;. \end{array}$$

For *path-sampling*, we use a definition of the reactive coordinate $\sigma_{ik}\left(t\right)$, given by:

(38) $$\sigma_{ik}\left(t\right)=L_{ik}\left(t\right),$$

with $L_{ik}\left(t\right)=\oint_{t}p_{ik}dq_{ik}$, equal to the path integral reached by integration until time t for the bias with index *i* and atom *k*. In Cartesian coordinates: $\sigma_{ik}\left(t\right)=\left\{L_{x_{ik}}\left(t\right),L_{y_{ik}}\left(t\right),L_{z_{ik}}\left(t\right)\right\}$ and $L\left(t\right)=\left\{\oint p_{x_{ik}}dx_{ik},\oint p_{y_{ik}}dy_{ik},\oint p_{z_{ik}}dz_{ik}\right\}$. Along $\sigma_{ik}$, a history-dependent bias potential $\Phi_{ik}^{t}$ is added in intervals of $\tau_{2_{ik}}$:

(39) $$\Phi_{ik}^{t}=-\frac{\partial }{\partial \sigma_{ik}}W_{ik}\sum_{t\leq t_{b}}\prod_{ik}\exp\left(-\frac{|\sigma_{ik}-\sigma_{ik}^{t-\tau_{2_{ik}}}|}{2\delta \sigma_{ik}^{2}}\right)\;,$$

where the height $W_{i}$ and the width $\delta \sigma_{i}$ are conventionally parameters chosen to provide computational efficiency and an efficient exploration of $F(s_{i})$. That formulation constantly drives the system to explore new configurations along the variable $L_{ik}$ and prevents the system from revisiting conformers which have been sampled previously. For the implementation of an efficient exploration of this space, we note that our algorithm uses the definition [17]:

(40) $$\delta \sigma_{ik}=\left|\sigma_{ik}^{t_{b_{ik}}}-\sigma_{ik}^{t_{b_{ik}}^{\prime }}\right|\;,$$

where the times $t_{b_{ik}}$ and $t_{b_{ik}}^{\prime }$ are defined by $\tau_{2_{ik}}=t_{b_{ik}}-t_{b_{ik}}^{\prime }$. We apply a variable height of each Gaussian added to an individual variable:

(41) $$W_{ik}=W\exp\left(-\Phi_{ik}/\Delta E\right)\times \frac{|\sigma_{ik}^{t_{b_{ik}}}-\sigma_{ik}^{t_{b_{ik}}^{\prime }}|}{\sigma_{ik}^{t_{b_{ik}}}}\;,$$

where *W* and ΔE are constants [96]. Finally, we mention that we introduce an identical renormalization of the *path-sampling* bias to the algorithm as we did for the PMF-enriched sampling. Therefore, we apply Equation (23) also to the bias *s*, as determined over the parallel path increments $dL_{ik}$, with a dependence on the coupling factor α.

### 2.11. Program and System Preparation

#### 2.11.1. PDB Data Collection and Data Processing

We applied in-house scripts in combination with visual MD (VMD) [97] plugins and the *mmLib*-Python library to access and analyze the PDB data [98]. For the selection of the PDB data for proteins, we excluded structures in which the proteins are bound to DNA or RNA and allowed for protein–protein complexes; we used only X-ray-resolved structures with a resolution higher than 2.5 Å and selected non-redundant sequences from the website http://www.rcsb.org. (We selected over 24,300 X-ray structures of proteins with a resolution <2.5 Å, where the sequence length is larger than 50 residues, no DNA or RNA is present in the system, and the set of structures contains less than 70% sequence identity. For DNA, we selected over 1800 X-ray structures with a resolution higher than 3.0 Å, containing DNA longer than 5 nucleotides, no RNA is present, and the structures are allowed to contain proteins longer than 20 amino acids.) DNA conformations of dinucleotide steps were assigned, and only structures with conformationally well-defined steps belonging to the 44 NtC classes were used. This procedure reduced over 20% of the noise contained in the DNA crystal structures. Currently, no comparable approach exists for the processing of protein structures. The analysis of the data was performed using in-house programs. The radial distribution functions g(r) and the p-PMF w(r) data was deposited into matrices as a function of the amino acid index and the distance *r*. The approximation of a *quasi*-homogeneity in the PDB dataset was implemented through the introduction of a constant number of atoms and a constant volume of $\frac{4}{3}\pi \times 2.5^{3}$ nm${}^{3}$. For the generation of the p-PMF data used in the simulation of proteins, an independent program analyzes the protein sequence and edits sequence-specific p-PMF data for the simulation of the backbone atoms (C, N, O, and Cα) belonging to protein chains. For the generation of the p-PMF data for the simulation of DNA, a version of the same program analyzes the DNA sequence and edits sequence-specific p-PMF data for the simulation of the nucleic acid atoms (P, N9/N1, C4/C2, C4’, C5’, and O5’) belonging to the DNA strands. The modified Gromacs routine *gmx_mdrun* (with main changes applied to the routine */src/kernel/md.c*) parses the p-PMF data and performs enhanced sampling using the methodology described in the Methods section. See the Supplementary Material for the analysis of the p-PMF data for both protein and DNA moieties.

#### 2.11.2. System Preparation and Simulation Parameters

We used the GROMACS package version 4.5.5 (www.gromacs.org) for the implementation of the methods and the analysis of the trajectories [10]. In more detail, we used the modules *gmx_pdb2gmx, gmx_editconf, gmx_genbox, gmx_grompp* for the setup of each system for the simulation. We used periodic boundary conditions in *x, y, z*. Each box was filled according to the normal density of water at room temperature. For the simulations of the dialanine peptide, we centered Ace-Ala-NMe into a cubic box with dimensions 2.26703×2.26703×2.26703 nm${}^{3}$ and filled the box with 371 SPC/E waters. We generated the extended peptide geometries (Ace-X-X-X-X-X-NMe, Ace-X-X-X-X-Y-NMe, Ace-X-X-X-Y-X-NMe, Ace-X-X-Y-X-X-NMe, Ace-X-Y-X-X-X-NMe, Ace-Y-X-X-X-X-NMe (X = Ala, Y = Ser)), and solvated each pentapeptide with 1184 SPC/E waters in a box with dimensions 3.32×3.32×3.32 nm${}^{3}$. We applied the parameters $\alpha =10^{-6}$ and ϵ=10.0 to the PMF-enriched simulations of the pentapeptides. For the simulations of TrpCage (NLYIQWLKDGGPSSGRPPPS) [73], we generated the starting structure using the *Ribosome* program and centered the extended peptide in a box with dimensions 7×7×7 nm${}^{3}$, filled the box with 11.424 SPC/E waters, and added one chloride ion. In the simulation of TrpCage, we used the AMBER99 SB force field. For the *path-sampling* simulations of TrpCage and the pentapeptides, we used the recently developed path-dependent enhanced sampling method [82] with coupling times $\tau_{1}$ = 850 fs and $\tau_{2}$ = 550 fs and a coupling factor α of $10^{-4}$ with ϵ = 25 as the fluctuation parameter for the renormalization to the unbiased gradient in the system [83] (see Methods section). We applied four different techniques to the folding simulations of TrpCage: *path-sampling*, the direct application of the p-PMF (direct-p-PMF), PMF-enriched sampling, and a combination of *path-sampling* and the PMF-enriched method. We simulated the *path-sampling* simulation of folding of TrpCage for 100 ns, the direct-p-PMF simulation for 225 ns, and the PMF-enriched simulation for 200 ns. In the setup of the DNA simulations, we centered the X-ray starting structure (PDB: 1bna) of the Dickerson–Drew DNA dodecamer d(CGCGAATTCGCG) [74] in a triclinic box with dimensions 3.64×4.099×5.83 nm${}^{3}$, then filled the box with 2615 SPC/E waters and added 22 sodium ions to neutralize the system. For the simulation of the Dickerson–Drew dodecamer, we used direct p-PMF (see Supplementary Material), PMF-enriched sampling, and standard MD simulations. We applied the PMF-based methodology using α parameters equal to $10^{-9}$ and ϵ parameters equal to 1 in all simulations. The PMF set was generated using the global PMF data depending on the DNA sequence. We used the AMBER versions 99, 12sb, and 14sb for the description of the interactions in the system [6,7]. For three PMF-based simulations (AMBER99, AMBER12sb, AMBER14sb), we used a coupling factor of $\alpha =10^{-9}$ and for another set of three PMF simulations (AMBER99, AMBER12sb, AMBER14sb), we used $\alpha =10^{-8}$.

In each simulation, we applied the Nosé-Hoover thermostat with a coupling time $\tau_{T}=1$ ps to simulate the system at a temperature equal to 300 K (*constant-NVT* ensemble). We used three different force fields to test the effect of the PMF-enriched method if an approximately identical partition as a function of the NtC index could be reached. We selected the coupling factor α equal to $10^{-9}$ with an upper value of $10^{-8}$, at which the stability of the double-helical DNA is not affected. We simulated the same DNA system with the same force field parameters for 100 ns with standard MD (time step of 2 fs), while the PMF-enriched simulations were simulated for 5 ns with a time step of 1 fs. Lennard-Jones and electrostatic interactions were calculated using a cutoff of 1.0 nm. We used the Particle Mesh Ewald (PME) algorithm for the calculation of the electrostatic interactions, and a shift function was used to calculate the vdW interactions. We used a neighbor list with a cutoff of 1 nm, which was updated every time step. We used a time step of 1 fs. The water geometry was kept rigid using the SETTLE algorithm, while no constraints were applied to the DNA except in the MD simulation, where we applied LINCS constraints to the hydrogen bonds of the Dickerson–Drew dodecamer [99].

For the analysis of the data, we used in-house programs in combination with analyses using *gmx_angle* for the measurement of dihedral angles, and *gmx_rms* and *gmx_gyrate* for the analysis of the folding of TrpCage. We used the *PyMOL* program for the visualization of the structures. For the measurement of the relative free energies ΔF, we used the relation:

(42) $$\Delta F=-k_{B}T\ln P\;,$$

where *P* stands for the probability of finding the system at a coordinate within a projected free energy landscape (FEL).

#### 2.11.3. TrpCage

TrpCage is a 20-residue-long synthetic peptide with a structure containing an N-terminal α-helix, a middle $3_{10}$-helical part, followed by a C-terminal polyproline helix. The central element is the stabilizing hydrophobic contact between the C-terminal Pro residues and a central tryptophan Trp6, while Trp6 is partially stacked with a neighboring tyrosine residue Tyr3 and stabilized by a salt-bridge between Arg16 and Asp9 [73].

#### 2.11.4. Dickerson–Drew DNA Dodecamer

Analysis of the synthetic, double-helical Dickerson–Drew DNA dodecamer (PDB: 1bna [74]) crystal structure reveals that only half of the steps adopt the canonical BI conformation. A smaller fraction of approximately 20% of the steps have B-A conformations, while 15% are in BII or mixed BI/BII configurations. The smallest fraction, 10%, of the dinucleotide steps adopt A and A-to-B forms [68]. Analogous to the broadly accepted structural heterogeneity of proteins, especially intrinsically disordered peptides, we observe that the DNA dodecamer accesses a larger number of classes, ranging from the A to the B form, which we characterized by a classification using the structural alphabet of DNA.

#### 2.11.5. Assignment of DNA Conformers with the Structural Alphabet for DNA

We used the module gmx_angle for the analysis of the dihedral angles. In Figure S1 in the Supplementary Material, we list the atom names used for the definition of the dihedral angles (see Figure S1). We used the set of 9 torsions for the definition of 44 different conformer classes based on the nucleotide conformer classes (NtC), as described in the references [66,67,68] (see also the webserver www.dnatco.org). The NtCs are described in the DNA structural alphabet, which describes structural polymorphisms of DNA through a classification of dinucleotide structures of 60,000 DNA steps from sequentially nonredundant crystal structures in an automated protocol assigning 44 distinct structural (conformational) classes, which are the so-called NtCs (for nucleotide conformers). The NtC assignment is applied to analyze the structural properties of typical DNA structures as they occur in our simulation trajectories. At present, no alternative method exists for the structural classification of DNA conformations. The NtC assignment represents a very defined approach to classifying DNA conformations and conformation partitions averaged over the simulations. (For the studied dodecamer, residues 1–12 form the first DNA strand (chain A in the 1bna) and residues 13–24 belong to the second strand (chain B). Within each strand containing 12 nucleotide residues, there can be assigned 11 dinucleotide steps numbered from 1 to 11 for the first strand and from 13 to 23 for the second. The step-index #N then corresponds to a dinucleotide formed by residues N and N + 1.)

**Figure 2:**
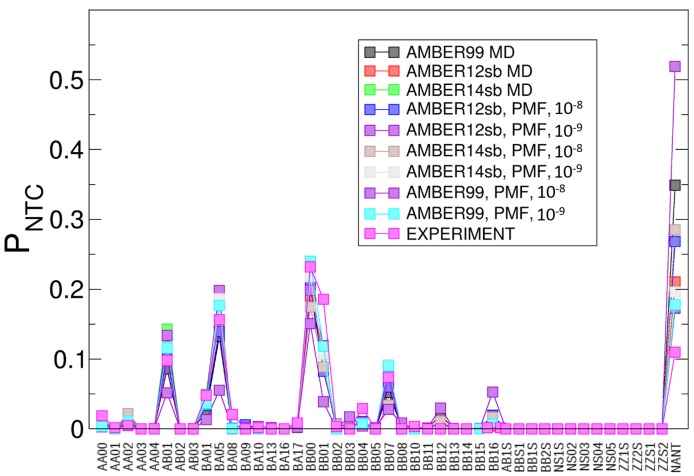
Probability *P* for the occurrence of a structural class in the 6 different PMF-enriched, 3 MD simulations (AMBER99, AMBER12sb, AMBER14sb, with 2 coupling factors α: $10^{-8}$ and $10^{-9}$), and a set of 29 experimental X-ray structures of the same dodecamer as a function of the class index. We used this analysis as the validation of the assignment of conformers and the technique using the auxiliary Hamiltonian H(B) in combination with a standard force field (the Hamiltonian H(A)). The experimental values for these conformers equal 15.6 (BA05), 23.2 (BB00), and 7.3% (BB07), which is in good agreement with the simulation result. Only for the non-assigned class (NANT) do we observe a larger variation in values, ranging from 10 to above 50%, where the experimental value equals 11%. We conclude that the populations of the NtC classes from the PMF-enriched simulations are in good agreement with the MD simulations and the experimental PDB structures of the same molecule, which underlines a key property of the method, namely, the increase in the accessible conformation space of DNA toward the partition described in the PDB.

In order to assess the quality of the conformations sampled by the PMF-enriched and the MD simulations, we compared the partitions of structural classes in the simulations and the experiment (see Figure 2). For the comparison with the experimental NtC partitions of the Dickerson–Drew DNA dodecamer, we selected 29 DNA structures of sequentially related dodecamers contained in the PDB: 1bna, 1d29, 1ehv, 1fq2, 1g75, 1g8n, 1n1o, 1z5t, 436d, 4bna, 4c64, 4i9v, 4pwm, 1fq2, 1g8u, 1ndn, 2bna, 455d, 4c5x, 4f3u, 4kw0, 7bna, 1g75, 1g8v, 1vte, 3bna, 463d, 4c63, 4hqi, 4mgw, 9bna.

#### 2.11.6. Kinetic Analysis and Analysis of Free Energy Partitions of DNA

We analyzed the time-dependent behavior of the DNA dodecamer and assigned each configuration to a set of classes along the sequence, resulting in 22 steps per time frame. For the determination of the transition frequencies $\nu_{ik}$, we analyzed the average time $\tau_{ik}$ needed for the system to change its state from one conformer class *i* to another conformation *k*. We used the logarithm of the inverse time to express the rate of transition, using:

(43) $$\ln\nu_{ik}=\ln\left(\frac{1}{\tau_{ik}}\right)\;.$$

## 3. Results and Discussion

### 3.1. Dialanine

**Figure 3:**
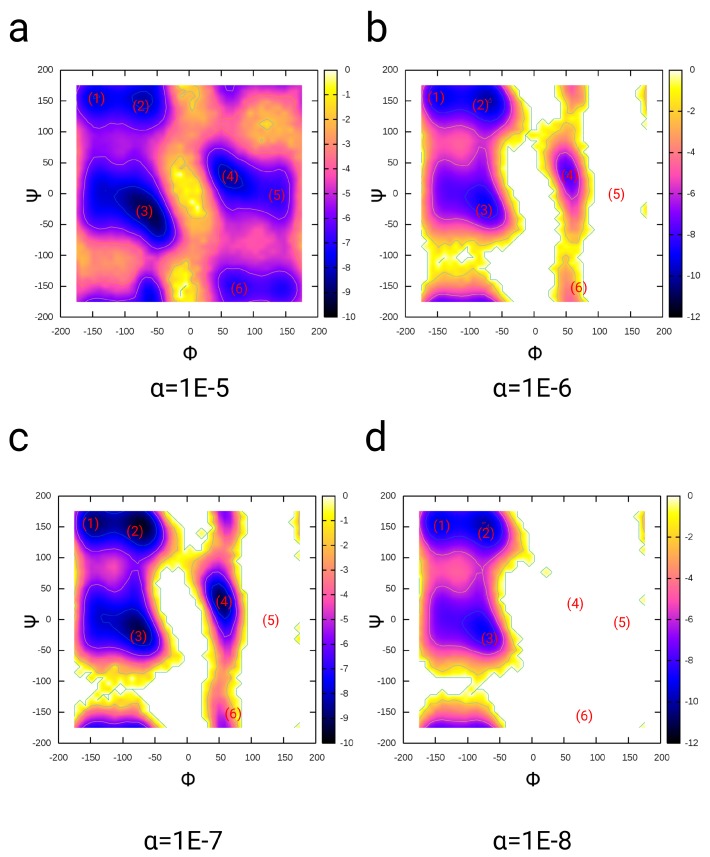
Free energy landscapes of dialanine as a function of dihedral angles Φ and Ψ for different coupling factors α ranging from $10^{-8}$ to $10^{-5}$ (panels: (**a**–**d**)). We simulated dialanine at room temperature (300 K) for 50 ns using the PMF-enriched sampling method (ϵ=20). The partitions are in agreement with 1 μs MD trajectories [83]. For the determination of the acceleration of the PMF-enriched methodology, we determined escape times τ of the Φ-angle (the transition time for Φ to change its value from <0 to values >0). We observe for $\alpha =10^{-5}$, τ=282.3 ps; for $\alpha =10^{-6}$, τ=2586.9 ps; for $\alpha =10^{-7}$, τ=3244.4 ps; and for $\alpha =10^{-8}$, τ=∞ (no transition). The relative acceleration *n* of the PMF-enriched method for $\alpha =10^{-5}$ equals a factor of n=151.2 (MD: τ=42696.7 ps [83]); for $\alpha =10^{-6}$, n=16.5; and for $\alpha =10^{-7}$, n=13.1. The colored bar in each of the free energy landscapes corresponds to energy values in units of $k_{B}T$.

For the validation of the PMF-enriched sampling, we chose dialanine as a test case, where we varied the α parameter and tested the convergence of the free energy partition along the order parameters Φ and Ψ (see Figure 3). Dialanine is a well-tested system for the validation of enhanced sampling algorithms [17,100,101] and has been used to study the kinetics of transitions along the reactive coordinates [102,103,104]. We simulated each system for 50 ns at 300 K using α parameters ranging from $10^{-8}$ to $10^{-5}$ with the parameter ϵ= 20. In the simulation using $\alpha =10^{-5}$, we observe populations at the angles $-150^{\circ }$ < Φ < 50${}^{\circ }$, 100${}^{\circ }$ < Ψ < 180${}^{\circ }$ (1, 2) (ΔF≈−8 $k_{B}T$), −100${}^{\circ }$ < Φ < −30${}^{\circ }$, −80${}^{\circ }$ < Ψ < 0${}^{\circ }$ (3) (ΔF≈−10 $k_{B}T$), 35${}^{\circ }$ < Φ < 150${}^{\circ }$, −20${}^{\circ }$ < Ψ < 80${}^{\circ }$ (4, 5) (ΔF≈−9 $k_{B}T$) and 50${}^{\circ }$ < Φ < 100${}^{\circ }$, −180${}^{\circ }$ < Ψ < −135${}^{\circ }$ (6) (ΔF≈−7 $k_{B}T$) (see Figure 3a). When we reduce the coupling by a factor of 10 to $\alpha =10^{-6}$, we measure partitions in the ranges of (1, 2) (ΔF≈−10 $k_{B}T$), and at (3, 4) we measure an approximately identical depth, while the minima at (5) and (6) disappear due to the lower amount of energy added by the p-PMF-derived Hamiltonian H(B) (see Figure 3b). With the factor $\alpha =10^{-7}$, we observe an optimal partition at the minima (1, 2), (3), and (4), with energy values equal to −10$k_{B}T$ (see Figure 3c), while we observe no transitions along the Φ-angle with the coupling constant $\alpha =10^{-8}$, where the added energy is too low to facilitate a change in dialanine along the Φ-angle order parameter (see Figure 3d). We determined an acceleration factor *n* through a comparison of the transition times τ along the Φ-angle from negative <0${}^{\circ }$ to positive values >15${}^{\circ }$ with 1 μs standard MD simulations [83]. We measured for parameter $\alpha =10^{-5}$, τ=282.3 ps; for $\alpha =10^{-6}$, τ=2586.9 ps; for $\alpha =10^{-7}$, τ=3244.4 ps; and for $\alpha =10^{-8}$, τ=∞ (no transition). As relative acceleration factors *n* of the PMF-enriched method, we obtain for $\alpha =10^{-5}$, n=151.2 (MD: τ=42,696.7 ps [83]); for $\alpha =10^{-6}$, n=16.5; and for $\alpha =10^{-7}$, n=13.1.

### 3.2. Penta-Alanine with Ser Mutations

We validated the PMF-enriched sampling method using simulations of penta-alanine and Ser mutations in this peptide at alternating positions along the sequence (see Figures S6–S13 and the section: ’*Penta-alanine configuration is dependent on the position of one Ser residue*’ in the Supplementary Material). In the path-sampling simulations of penta-alanine, we observe the formation of an α-helical conformation, which shifts depending on the position of Ser in the mutated peptides, where the strongest change from the compact α-helical configuration is observed when Ser is in the N-terminal position. The PMF-enriched simulations contain the same conformations as those in the path-sampling result, but we find shifts in the conformational landscape due to the broadened averaging over all possible secondary structure elements in the determination of the p-PMFs, while the shifts according to the Ser position in the sequence have the same tendency as in the path-sampling result. As we mentioned in the Methods section, the deviation from the partition can be caused by the averaging over a broad ensemble of structures containing secondary structures which are not part of the conformational partition of the system of interest. A general solution to that problem is the guided and system-dependent selection of structures.

### 3.3. Simulations of TrpCage

#### 3.3.1. Path-Sampling Simulations

**Figure 4:**
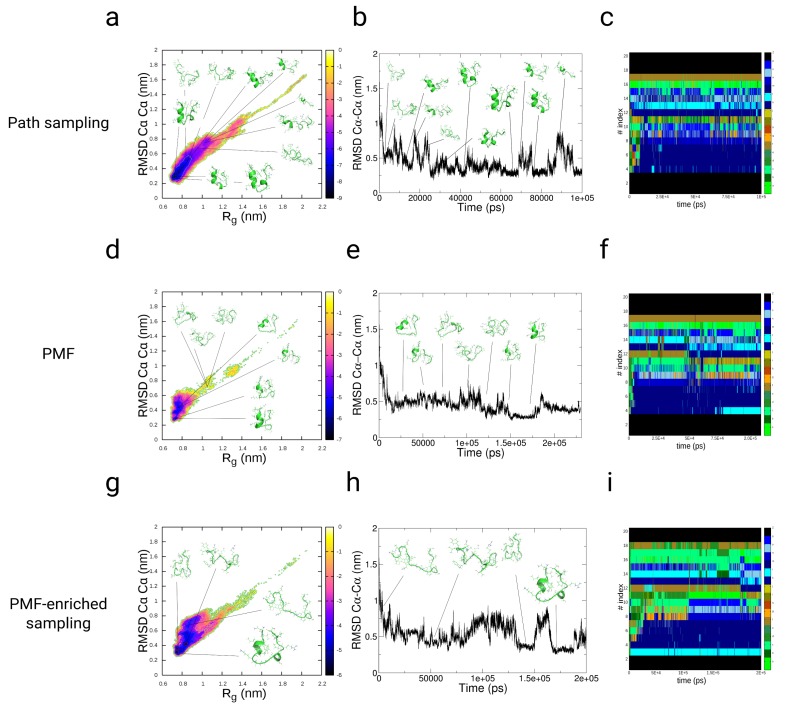
Results from adaptive *path-sampling* and PMF-enriched sampling simulations of TrpCage [73]. (Results from *path-sampling* (**a**–**c**), the direct application of p-PMFs without partitions $\Omega (r_{ij})$ and $\Omega (\theta_{k})$ (**d**–**f**), and PMF-enriched sampling (**g**–**i**)). We started the simulation from an extended structure in explicit SPC/E water. (**a**,**d**,**g**) Free energy landscape (FEL) as a function of $RMSD_{C\alpha -C\alpha }$ to the backbone of the native structure (PDB: 1l2y) and the radius of gyration Rg. Conformers shown in the FEL are the main conformations as observed in the cluster analysis of the trajectory. (**b**,**e**,**h**) $RMSD_{C\alpha -C\alpha }$ to the backbone of the native structure (PDB: 1l2y) as a function of the MD simulation time. (**c**,**f**,**i**) Assignment of conformers along the peptide to the structural alphabet of proteins as a function of simulation time and the sequence index of TrpCage [105]. The application of PMF-enriched sampling increases the folding time by a factor of approximately 10 compared to that observed from *path-sampling*. In contrast to the direct application of the p-PMF and the *path-sampling* method, the number of conformations near the folded minimum is larger, leading to the occurrence of three maxima, which might play a role in the near-native conformers not being described by the underlying force field. We note that we see a clear difference in the direct application of the p-PMFs to the protein and the propagation using the partition function Ω, where the associated error of the p-PMFs is compensated.

We applied the enhanced sampling method, which accelerates the system-dependent sampling on a path variable *L* [83]. Using this method for the folding of the TrpCage peptide, we observe a main population along the FEL as a function of the $RMSD_{C\alpha -C\alpha }$ and the radius of gyration (Rg) in the range $0.23<RMSD_{C\alpha -C\alpha }<0.28$ nm and 0.68<Rg<0.75 nm at an energy value of −9 $k_{B}T$ (see Figure 4a). This minimum is located within a low-energy region of −7 $k_{B}T$, which we find in the range $0.22<RMSD_{C\alpha -C\alpha }<0.45$ nm and 0.66<Rg<0.84 nm. We observe a third, broader region defined by $0.20<RMSD_{C\alpha -C\alpha }<0.8$ nm and 0.66<Rg<1.00 nm associated with an energy value of −5 $k_{B}T$, followed by the region $0.60<RMSD_{C\alpha -C\alpha }<1.1$ nm and 1.10<Rg<1.40 nm at an energy ranging from −3 to −4 $k_{B}T$. The region above $RMSD_{C\alpha -C\alpha }>1.1$ nm is only populated by the system at the initial stage of the simulation. The folding pathway of the peptide starts with the formation of a bend in the region from Lys8 to Gly10 at t = 3.8 ns, followed by a coiled structure at 6.3 ns at which point Leu7, Arg16, Asp9, and Pro18 are interacting non-specifically. At a simulation time of 12 ns, we observe the formation of an α-helix between residues Asn1 and Pro12, which corresponds to the native N-terminal α-helix. After that event of helix formation, we observe the occurrence of two jumps in the $RMSD_{C\alpha -C\alpha }$ from ≈0.36 nm to a value >0.7 nm, and we observe a collapse of the peptide to $RMSD_{C\alpha -C\alpha }<0.25$ nm at 23 ns. Within the remaining simulation time, we observe a population of states in the range of $0.2<RMSD_{C\alpha -C\alpha }<0.8$ nm, while the system mostly resides in the range $0.2<RMSD_{C\alpha -C\alpha }<0.4$ nm (see Figure 4b). Based on our result obtained from the simulations of dialanine, we determined an acceleration factor α equal to 154.6 for this protein from the *path-sampling* simulations [83]. Therefore, the total simulation time corresponds to $\tau =100\times 10^{-9}\;s\times 154.6\approx$15.4 μs, while the first folding event to $RMSD_{C\alpha -C\alpha }<$0.3 nm occurs at 3.54 μs. In the assignment analysis of conformers to the structural alphabet of proteins, we observe populations of helical conformers for the residues 4–8 (conformer k), while residues 9–11 populate conformations p, c, and g (helical and coil partially extended) and amino acids in the range of 12–17 reside in range of conformers k, l, a/c, and i (see Figure 4c).

#### 3.3.2. Folding of TrpCage: Direct p-PMF Simulations without Partitions $\Omega (r_{ij})$ and $\Omega (\theta_{k})$

As a test case, we applied the p-PMFs directly, without redefining the gradient using the partition functions Ω, starting from the same extended conformation as in the *path-sampling* simulation (direct p-PMF). In the free energy landscape as a function of the $RMSD_{C\alpha -C\alpha }$ and the radius of gyration Rg, we observe a main population along the FEL as a function of the $RMSD_{C\alpha -C\alpha }$ and the radius of gyration (Rg) in the range $0.23<RMSD_{C\alpha -C\alpha }<0.28$ nm and 0.68<Rg<0.75 nm at an energy value of −7 $k_{B}T$ (see Figure 4d). This minimum is located within a low-energy region from −4 to −5 $k_{B}T$, which we find in the range $0.22<RMSD_{C\alpha -C\alpha }<0.6$ nm and 0.6<Rg<0.8 nm. We observe a third region within $0.20<RMSD_{C\alpha -C\alpha }<0.8$ nm and 0.66<Rg<1.1 nm associated with an energy value of −1 $k_{B}T$. The region above $RMSD_{C\alpha -C\alpha }>0.8$ nm is only populated by the system at the initial stage of the simulation. The population densities differ between the *path-sampling* and the PMF-enriched simulation. The folding pathway of the peptide starts with the formation of a bend in the region from Lys8 to Gly10 at the initial stage of the simulation (1–2 ns), followed by helix formation between residues 1 and 8 (approximately 10 ns), where we do not observe the formation of the $3_{10}$-helical part between residues Gly10 and Ser14 (extended conformation). In a simulation time ranging from 25 to 50 ns, the contact between Pro19 and Tyr3 breaks, leading to an opening of the structure, while the α-helix remains conserved. We observe a long period of fluctuations between $RMSD_{C\alpha -C\alpha }\approx \;0.5$ nm and $RMSD_{C\alpha -C\alpha }\approx \;0.75$ nm in the time ranging from 50 to approximately 150 ns (see Figure 4e). All of the visited states contain an open conformation between the N-terminal α-helix and the C-terminal part. After the fluctuating period, we observe a collapse to $RMSD_{C\alpha -C\alpha }<0.25$ nm, where the $3_{10}$-helical part forms simultaneously with the poly-Pro and the N-terminal α-helical segment at 150 ns. The peptide resides in this state until 180 ns, when the peptide returns to the partially unfolded state with a partially open conformation between the N-terminal α-helix and the C-terminal part. In the assignment of conformers to the structural alphabet of proteins, we find populations of helical conformers for the residues 4–8 (conformers k, l), while residues 9–11 populate conformations p, c, and g (helical and coil partially extended) and amino acids in the range from 12 to 17 are designated as conformers k, l, a/c, and i (see Figure 4f). In contrast to the *path-sampling* simulation, the occurrence of changes in each of the protein blocks is higher, which is expressed by a higher propensity for changes in the patterns for residues 4–8, 9–11, and 12–17. We conclude that some of the conformers are connected to errors introduced by the direct application of the p-PMFs, which contain shifts in the energies due to the assumption of data homogeneity. As we find from the PMF-enriched simulation, the PMF-enriched approach compensates for the errors and also significantly broadens the conformational space.

#### 3.3.3. Folding of TrpCage: PMF-Enriched Simulations

As a final example, we applied the PMF-enriched method to folding simulations of the same peptide, where we applied the partitions $\Omega (r_{ij})$ and $\Omega (\theta_{k})$ to correct the gradient and remove the potential error introduced through the assumption of a homogeneity in the data. In the free energy landscape as a function of the $RMSD_{C\alpha -C\alpha }$ and the radius of gyration Rg, we observe the main population along the FEL as a function of the $RMSD_{C\alpha -C\alpha }$ and the radius of gyration (Rg) in the range $0.23<RMSD_{C\alpha -C\alpha }<0.45$ nm and 0.68<Rg<0.9 nm at an energy value of −6 $k_{B}T$, which shows a clear broadening of the minimum compared to the direct application of the p-PMF and the *path-sampling* result (see Figure 4g). This minimum is located within a low-energy region from −4 to −5 $k_{B}T$, which we find in the broad range $0.22<RMSD_{C\alpha -C\alpha }<0.8$ nm and 0.6<Rg<1.0 nm. We observe a third region within $0.20<RMSD_{C\alpha -C\alpha }<0.9$ nm and 0.66<Rg<1.2 nm associated with an energy value from −1 to −2 $k_{B}T$. The region above $RMSD_{C\alpha -C\alpha }>0.8$ nm is only populated by the system at the initial stage of the simulation. The population densities differ in this simulation from the first two simulations, where we find three main maxima at $RMSD_{C\alpha -C\alpha }=0.25$ nm, Rg=0.7 (1), $RMSD_{C\alpha -C\alpha }=0.3$ nm, Rg=0.7 nm (2), and $RMSD_{C\alpha -C\alpha }=0.4$ nm, Rg=0.85 nm (3). The folding pathway of the peptide occurs on a slower timescale and starts with the formation of a large number of unfolded intermediates, while a bend in the region from Lys8 to Gly10 is formed (1–50 ns). The helix-formation between residues 1 and 8 occurs at approximately 130 ns, while we observe also the formation of the $3_{10}$-helical part between residues Gly10 and Ser14 (see Figure 4h,i). In a simulation time range from 175 to approximately 185 ns, we observe a sharp decay in the RMSD to values lower than 0.25 nm and the formation of the folded state. We note that the folding process itself is strongly impeded by the application of conformational enrichment by the p-PMF partition, and the folding time is approximately 10 times larger than in the *path-sampling* simulation. In contrast to the direct application of the p-PMF and the *path-sampling* method, the number of conformations near the folded minimum is larger, leading to the occurrence of three maxima, which might play a role in the near-native conformers not being described by the underlying force field. We note that we see a clear difference between the direct application of the p-PMFs to the protein and the propagation using the partition function Ω, where the associated error of the p-PMFs is compensated. However, another effect from the p-PMF averaging is visible as we observed in simulations of penta-alanine and Ser-mutated versions of that peptide. The sequence-dependent PDB data from which the p-PMFs have been derived contains various secondary structural forms, which differ from α-, $3_{10}$-, and Poly-proline-helical structures, such as coiled coils or even β-stranded conformations, leading to deviations along the folding pathway. Although the renormalization of the Hamiltonian and the generation of the hybrid Hamiltonian are efficient, a proportion of minima in the p-PMFs arising from other secondary structural elements guides the system toward partially stretched conformations, which might not be part of the secondary structure of TrpCage along its folding pathway. As we have mentioned in the Methods section, a guided generation of p-PMF data will largely improve the conformational sampling, e.g., for TrpCage, PDB structures should be selected with an approximately similar number of atoms, as well as with α- and $3_{10}$-helical secondary structural content, but no β-stranded elements.

#### 3.3.4. Discussion

In the *path-sampling* simulations of the folding of TrpCage, we observe an initial formation of the $3_{10}$-helix, followed by a fast collapse to the native state with the formation of the N-terminal α-helix and the closure between the N- and the C-terminal segments of this peptide. In contrast, the direct p-PMF and the PMF-enriched simulations show a slower process of helical formation and closure of the tertiary structure than in the *path-sampling* simulation. In both PMF simulations, the unfolded state is populated in the PMF-enriched simulation, in general, for 4 times longer than in the *path-sampling* simulation of TrpCage. However, the PMF-enriched simulation results in a broader free energy partition with a larger heterogeneity of states close to the native minimum of this protein. In the *path-sampling* simulations, we identify the formation of the $3_{10}$-helix as first step prior to the formation of parts of the α-helix of this protein. In contrast to that observation, both PMF-coupled simulations show a larger residence time in the unfolded region and an initial formation of the α-helical part. In general, a folding pathway involving a helix-rich structure is in agreement with experiments [106,107] and other simulations [107,108,109,110,111,112]. In the PMF-enriched simulations, we observe a long-lived population of the unfolded ensemble at $RMSD_{C\alpha -C\alpha }$ values >0.5 nm, which might be equivalent to a molten globule state, as found in previous simulations and experiments [110,113,114]. A guided and system-dependent selection of PDB structures for the generation of p-PMF data can lead to improvements in the quality of the sampling of structural conformers. In this study, we tested the case in which a broad PDB population is used for the PMF-enriched sampling, leading to quantitative convergence to the native state, although deviations in the conformation space of TrpCage are visible. A system-dependent selection of PDB structures can have a large impact on the folding times and visible secondary structures of TrpCage. Timescales of folding observed in the *path-sampling* simulation are in agreement with results from experiments [114], simulation results [115], and three independent MD/kMC results using our prior developments based on discrete moves and an adaptive kMC/MD method [83,116,117]. Taking the same formalism in the form of a linear acceleration factor, the kinetics in the PMF-enriched simulation show that the processes of folding occur 6–10 times slower than in the *path-sampling* simulation. The analysis using the structural alphabet for proteins shows a higher heterogeneity in the occupation of states in the PMF-enriched simulation than in the *path-sampling* simulation, which indicates that the use of PMF enrichment induces a population of a larger number of states than in the simulation using *path-sampling*.

### 3.4. Simulations of the Dickerson–Drew DNA Dodecamer

**Figure 5:**
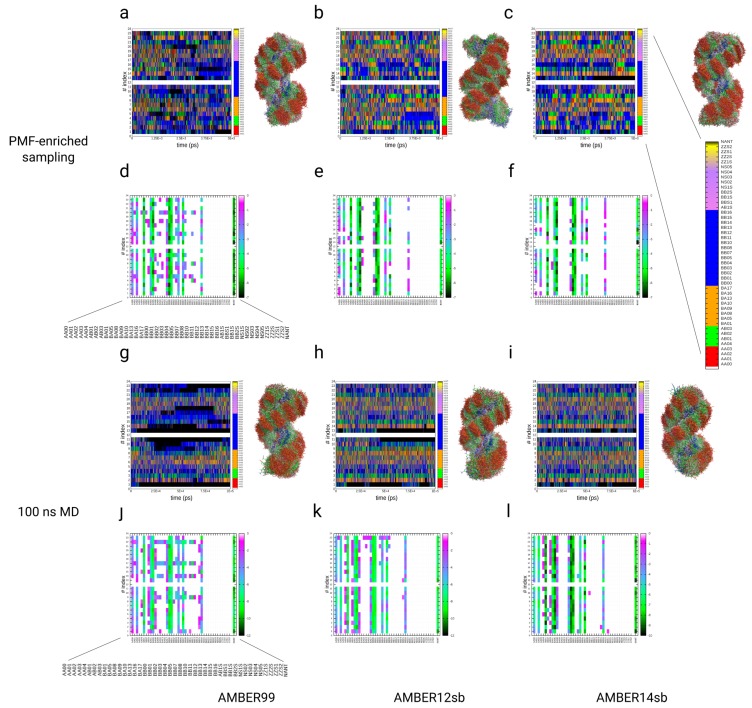
Results from the nucleotide conformer (NtC) assignment analysis of three PMF-enriched simulations of the Dickerson–Drew DNA dodecamer (with the coupling parameter $\alpha =10^{-9}$) (**a**–**f**) (see Methods section). The overlaid structures of each trajectory are displayed on the right side of the panels (**a**–**c**) and (**g**–**i**). We compare the data with 100 ns MD simulations of the same systems with identical force field parameter sets (**g**–**l**). (**a**–**c**,**g**–**i**) NtCs as a function of time from the simulation using different conventional force fields (H(A)): AMBER99 (**a**,**d**,**g**,**j**), AMBER12sb (**b**,**e**,**h**,**k**), and AMBER14sb (**c**,**f**,**i**,**l**). (**d**–**f**, **j**–**l**) Free energy partition ($\Delta F=-k_{B}T\ln\left(p/p_{min}\right)$) as a function of the conformer class index and the step-index along the DNA sequence from the simulations. The colored bar expresses the energies in units of $k_{B}T$.

**Figure 6:**
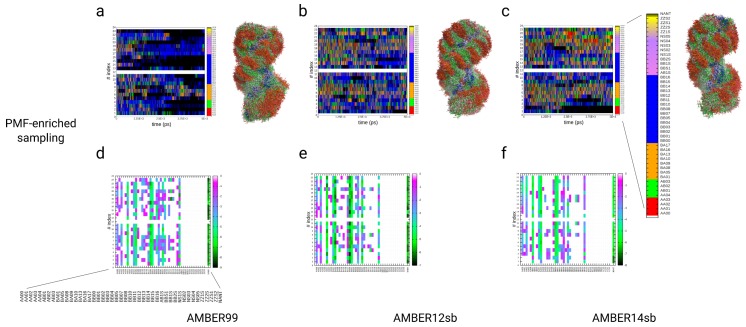
NtC assignment analysis of three PMF-enriched simulations of the Dickerson–Drew DNA dodecamer (with a stronger coupling parameter $\alpha =10^{-8}$) (see Methods section). The overlaid structures of each trajectory are displayed on the right side of the panels (**a**–**c**). (**a**–**c**) NtCs as a function of time from the simulation using different conventional force fields (H(A)): AMBER99 (**a**), AMBER12sb (**b**), and AMBER14sb (**c**). (**d**–**f**) Free energy partition ($\Delta F=-k_{B}T\ln\left(p/p_{min}\right)$) as a function of the conformer class index and the step-index along the DNA sequence from the simulations using AMBER99 (**d**), AMBER12sb (**e**), and AMBER14sb (**f**). The colored bar expresses the energies in units of $k_{B}T$.

**Figure 7:**
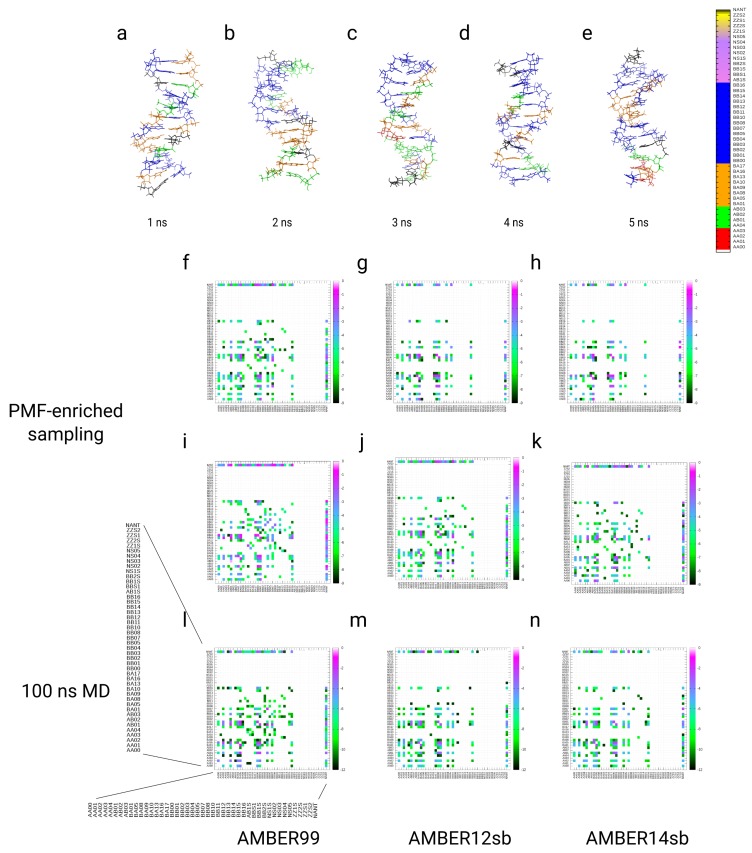
(**a**–**e**) Conformers of the Dickerson–Drew DNA dodecamer along the enhanced sampling trajectory over 5 ns using the AMBER12sb force field (with a coupling factor $\alpha =10^{-9}$). Conformers are color-indexed depending on their assignment to the 44 different NtC classes from the structural alphabet. The colored bar represents the structural classes shown on the right side. (**f**–**k**) Results from the kinetic analysis of the frequencies lnν, ν=1/τ of the transition time between different structural classes (in the order from class n shown on the x-axis to classes n’ on the y-axis) as a function of 44 different NtC classes in the PMF-enriched (**f**–**k**) and 100 ns MD simulations (**l**–**n**) (class #45 represents the non-assigned class)) (using the two different α coupling factors: $10^{-9}$ (**f**–**h**) and $10^{-8}$ (**i**–**k**)). We compared the three simulations with different force fields: AMBER99 (**f**,**i**,**l**), AMBER12sb (**g**,**j**,**m**), and AMBER14sb (**h**,**k**,**n**).

As a second example, we applied the PMF-enriched sampling on the Dickerson–Drew DNA dodecamer. In the MD and PMF-enriched simulations, we used three different force fields to test whether the PMF-enriched result reaches an approximately identical partition as a function of the NtC index and can describe approximately the same averages as in the MD simulations. For the analysis, we applied the assignment of NtC classes and compared the partitions, populations, and kinetics of transitions between classes with 100 ns MD simulations using three different AMBER force fields: 99, 12sb, and 14sb (see Figure 2 and Figure 5, Figure 6 and Figure 7). We also directly coupled the p-PMFs of DNA to the system without the application of the partition to investigate the impact of the additional re-evaluation with the partition function over the p-PMFs (see Figures S4 and S5). The direct application of the p-PMFs to the sampling without a redefinition in a partition leads to a larger deviation between the different simulations with different force fields, especially in the simulation with AMBER14sb. In the analysis of the PMF-enriched simulations, we investigated the time-dependent occurrences and the partitions of NtC classes for the simulation time with coupling factors $\alpha =10^{-9}$ and $\alpha =10^{-8}$. In the analysis of the partitions for the simulation time with $\alpha =10^{-9}$, we observe major populations of minima at the NtC classes BB00 and BB01 (ΔF≈−7 $k_{B}T$) for all three different force fields, with an approximately homogeneous partition over all step-indices (see Figure 5a–f). Within a variation of approximately 1 $k_{B}T$, the classes BA01 and BA05 are also populated with the same energies from approximately −5 to −6 $k_{B}T$, while we observe variations for the steps 10–11 and 13–14. The classes AB01 and BB07 are also populated with approximately identical patterns in each of the three force fields with energies from −4 to −5 $k_{B}T$, while we observe variations for BB07 at the step-indices 5–8 and 17–18 (see Figure 5a–f). The classes AA00, AA02, BA04, BA17, and BB16 again contain approximately identical energy values from −1 to −2 $k_{B}T$ (within variations of ±1 $k_{B}T$), where we find that these classes are less probable for the sequence of the Drew–Dickerson DNA dodecamer. For the MD simulations of the same dodecamer with the identical force field parameters, we also observe approximately identical patterns in the partitions over the NtC classes (see Figure 5g–l). As for the PMF-enriched simulations, we find the strongest population of the NtC classes BB00 and BB01 (−10 $k_{B}T$) in all three cases, while we find a slightly higher occurrence for the AMBER14sb simulation (ΔF≈ −12 $k_{B}T$). The classes BA01 and BA05 are populated with the same weight, while the classes AA00, AA02, BA17, BB04, and BB16 are associated with energies ranging from −2 to −6 $k_{B}T$ (with variations for the AMBER14sb simulations with approximately ± 1–2 $k_{B}T$). In general, the patterns of the partitions are approximately identical in the MD and the PMF-enriched simulations at $\alpha =10^{-9}$. We increased the coupling α to a value equal $10^{-8}$ and applied it to the same systems with identical force field parameters (see Figure 6a–f). The elevated energy added to the system facilitates the population of a broader spectrum in the space of NtC classes, while the strongest populations remain at the two classes BB00 and BB01 at energies ranging from −7 to −9 $k_{B}T$. We also observe approximately identical patterns for the classes BA01, BA05, AB01, BA17, AA00, AA02, and BB16, while, additionally, the classes AA01, BA09, BA10, BA13, BA16, BB02, BB03, and BB11–BB13 are populated at energies ranging from −2 to −4 $k_{B}T$ (see Figure 6a–f), which is an effect from the PMF enrichment of conformations at a higher coupling value. The elevated energies lower the barrier for transitions, especially between AA and BB forms, so that increased numbers of BA and AB classes are populated by the DNA dodecamer. At the same time, the location of the deepest minima along the partitions are identical with the MD result. In general, we observe that the transitions along the dodecamer are concerted along the double helix, so changes in the middle segments always involve correlated conformation changes in the terminal regions (see Figure 5a–c,g–i, Figure 6a–c and Figure 7a–e).

The analysis of the kinetic patterns of the transitions between the NtC classes averaged over the complete sequence of the dodecamer shows a quasi-symmetric picture of transitions between AA and BB forms, while the pathways differ between different intermediates represented by BA and AB classes in the MD and the PMF-enriched simulations (see Figure 7f–n). In general, the patterns of transitions between AA and BB forms are approximately identical in the MD and the PMF-enriched simulations, while transitions in the PMF-enriched simulations are faster by a factor equal to approximately 20.0 when we compare the relative magnitude of the transition frequencies (see Figure 7f–n). We state that we can consider the transitions from BB to AA forms as reversible reactions, with quasi-equilibria between BB and BA, AB forms, as well as reversible transitions between AA to AB and BA forms. Each of the quasi-reversible transitions contains the AA and BB forms as quasi-product states in the partition of transitions. Z-forms and *syn*-conformers, which occur preferentially in RNA but not DNA, are neither observed in our MD nor in the PMF-enriched simulations. As general features in the transition partitions, we find that the AA00 conformer is reversibly linked to AA02, AA04, AB01, AB03, BA01, BA05, BA08, BB00, BB01, and BB16 in the AMBER99 simulation, using $\alpha =10^{-9}$. In the two simulations with AMBER12sb and AMBER14sb with the same coupling strength, the conformer AA00 is reversibly linked to AA02, AB01, BA01, BA04, and BB00 (see Figure 7f,g,h). We also find that the conformer BB00 is linked to a change toward approximately the same conformations: AA00, AA02, AB01, AB03, and the BA conformers BA01, BA05, BA08, BA17, BB01, BB04, BB07, and BB16. In the MD simulations, the conformer AA00 is coupled to AA02, AB01, BA01, BA05, BB00, BB01, and BB16 in the AMBER99 simulation. The two other simulations with AMBER12sb and AMBER14sb show a reversible linkage of AA00 to approximately the same NtC classes. In the three simulations, the BB00 conformer is linked to AA00, AA02, AA04, AB01, AB03, BA01, BA05, BA08, BA17, BB01, BB04, BB07, and BB16 (see Figure 7l–n). In conclusion, we find that AA00 and BB00 conformers are reversibly linked through transitions within a larger number of BA and AB forms, while we note that the general patterns of transitions are approximately identical in the MD simulations and the PMF-enriched results. That result shows that transitions between the conformer classes AA and BB can occur via a larger number of up to 12 different pathways through reversible transitions. We observe also direct transitions between the conformers AA and BB, while we speculate that defined neighboring conformer classes lead to preferential populations of intermediates in a transition of a defined step. Although we find a large number of possible pathways for the transition between the AA and BB forms, we note that there might be potentially preferred pathways depending on the conformations within the complete DNA molecule.

We compared the relative populations of each PMF-enriched and MD simulation with experimentally observed partitions in X-ray structures (see Figure 2). The class AA00 is populated by 0.3–1.4% in the simulations, while the experimental partition yields a value equal to 1.9%. We observe an initial maximum for the conformer AB01, where the results are also in a close range with values from 5 to 13%, while the experimental value equals 9.8%. We observe further maxima for the conformers BA05, BB00, and BB07 with similar values of approximately 15, 20, and 7%. The experimental values for these conformers equal 15.6 (BA05), 23.2 (BB00), and 7.3% (BB07), and these are in good agreement with the simulation result. Only in the case of the non-assigned class (NANT) do we observe a larger variation in values, ranging from 10 to above 50%, where the experimental value equals 11%. While there are significant differences in the conformations sampled by classical MD using different force fields, we conclude that the different force fields reach approximately the same average as a function of the NtC-index using the PMF-enriched simulations. Quantitatively, the PMF-enriched simulation in combination with the AMBER99 force field yields the optimal result. We also note that the limited number of 29 X-ray structures of the DNA dodecamer as a reference can also potentially lead to errors compared with the real partition of conformations of that molecule. That could also be the cause for a lower propensity of NANT classes in that partition. Alternatively, the higher abundance of NANT classes in the dynamical simulation compared to the crystal structures could be connected with the structural states representing transitions between existing NtC classes, which would correspond with various experimental low-energy conformations. We conclude that the populations of NtC classes from the PMF-enriched simulations are in good agreement with the MD simulations and the experimental PDB structures of the same molecule, which underlines the potential of the novel method to improve the sampling toward a partition derived from the PDB, if the associated error δw is sufficiently low.

## 4. Conclusions

**Figure 8:**
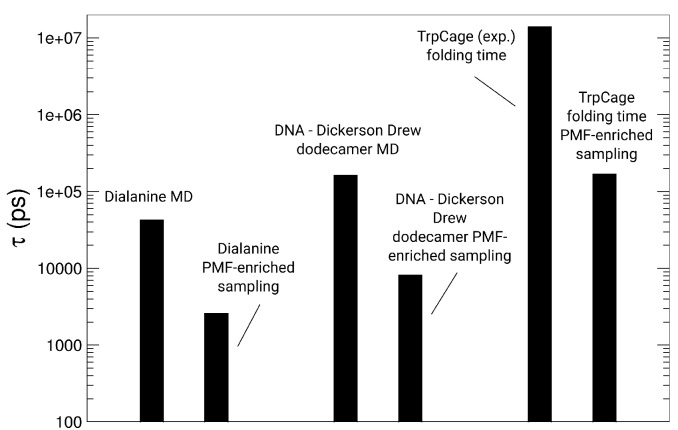
Comparison of timescales τ for relevant transitions of the systems simulated in this study with the PMF-enriched sampling technique: dialanine, the Dickerson–Drew DNA dodecamer, and TrpCage (experimental folding times ranging from 1.6 to 4 μs [113,114,116]). In each of the cases, we observe a significant acceleration of the PMF-enriched sampling technique compared with standard MD and experiments. For dialanine, we determine an acceleration by a factor ranging from n=13.1 to n=16.5. For the Dickerson–Drew DNA dodecamer, we measure that the PMF-enriched sampling is faster by a factor n=20.0, while for TrpCage, we determine a factor of approximately n=82.3 in comparison with the experiment.

In this article, we present a method for an enhanced molecular dynamics simulation of protein and DNA systems called potential of mean force (PMF)-enriched sampling. In general, the method applies partitions derived from potentials of mean force (PMFs), which we determined from DNA and protein structures in the Protein Data Bank. The technique enriches the conformational space of a DNA or protein molecule in the MD simulation with structural partitions from the PDB and accelerates transitions through the definition of a hybrid Hamiltonian consisting of the underlying force field and a Hamiltonian derived from the PDB partition. For this approach, we derived effective partitions over *pseudo*-potentials of mean force (p-PMF) from the Protein Data Bank (PDB), since the PDB structures resemble a large number of different Hamiltonians with different salt-concentrations, volumes, pressures, and temperatures, which makes the direct determination of potentials of mean force difficult. We solved that problem through the introduction of an approximation of *quasi*-homogeneity within the collected data and the definition of an error estimate that we introduced through the approximation procedure and defined the partition function of the p-PMFs, from which we derived the bias applied to the systems. We validated the method using simulations of dialanine, the folding of TrpCage, and the conformational sampling of the Dickerson–Drew DNA dodecamer (see Figure 8). Our results show the potential for the PMF-enriched simulation technique to enrich the conformational space of biomolecules along their order parameters, while we also observed considerable speed increase in the sampling by factors ranging from 13.1-16.5 (dialanine) to 82 (TrpCage). We compared the partitions over the NtC conformer classes of the structural alphabet of DNA from our simulations with the average obtained from experimental structures from the PDB. The PMF-enriched simulation technique can effectively be combined with enhanced sampling or coarse-graining methodologies for the improved sampling within a partition derived from the PDB.

## Supplementary Materials

The following are available online at http://www.mdpi.com/1422-0067/19/11/3405/s1. Figure S1: Dihedral angle definitions for the assignment of DNA-configurations to the structural alphabet of DNA. Figure S2: Results of the *pseudo* potential of mean force (p-PMF-w(r)) data-analysis of protein data bank (PDB) for peptides and proteins. Figure S3: Results from the data-collection over 1,800 X-ray structures of DNA in the PDB. Figure S4: Results from the assignment-analysis of the 3 trajectories of the Dickerson-Drew DNA dodecamer using the *pseudo* potentials of mean force directly without the definition of the corresponding partitions $\Omega_{i}$ with a coupling strength α of $10^{-4}$. Figure S5: Kinetic analysis of the 3 trajectories of the Dickerson-Drew DNA dodecamer using the *pseudo* potentials of mean force p-PMF directly without the definition of the corresponding partitions $\Omega_{i}$ with a coupling strength α of $10^{-4}$. Figure S6: Results from path-sampling and PMF-sampling simulations of the pentapeptides. Figure S7: Results from path- and PMF-sampling simulations of Penta-alanine Ace-Ala-Ala-Ala-Ala-Ala-NMe using the AMBER99 forcefield. Figure S8: Results from path- and PMF-sampling simulations of Penta-alanine with one Ala-Ser mutation: Ace-Ala-Ala-Ala-Ala-Ser-NMe using the AMBER99 forcefield. Figure S9: Results from path- and PMF-sampling simulations of Penta-alanine with one Ala-Ser mutation: Ace-Ala-Ala-Ala-Ser-Ala-NMe using the AMBER99 forcefield. Figure S10: Results from path- and PMF-sampling simulations of Penta-alanine with one Ala-Ser mutation: Ace-Ala-Ala-Ser-Ala-Ala-NMe using the AMBER99 forcefield. Figure S11: Results from path- and PMF-sampling simulations of Penta-alanine with one Ala-Ser mutation: Ace-Ala-Ser-Ala-Ala-Ala-NMe using the AMBER99 forcefield. Figure S12: Results from path- and PMF-sampling simulations of Penta-alanine with one Ala-Ser mutation: Ace-Ser-Ala-Ala-Ala-Ala-NMe using the AMBER99 forcefield. Figure S13: Summary of PMF-sampling simulations for penta-alanine and the mutated versions from the path sampling simulation.
