# Supplementary material for: Enriched Conformational Sampling of DNA and Proteins with a Hybrid Hamiltonian Derived from the Protein Data Bank
Source: Int J Mol Sci. 2018 Oct 30;19(11):3405. doi: 10.3390/ijms19113405 (PMC6274895; doi:10.3390/ijms19113405)
Supplement: Supplementary file 1 [file ijms-19-03405-s001.pdf]

International Journal of
*Molecular Sciences*

MDPI

*Article*

# Enriched conformational sampling of DNA and proteins with a hybrid Hamiltonian derived from the Protein Data Bank - Supplementary Material

**Emanuel K. Peter † * and Jiří Černý † ***

† Institute of Biotechnology of the Czech Academy of Sciences, BIOCEV, Průmyslová 595, 252 50 Vestec, Prague West, Czech Republic

* Correspondence: petere@ibt.cas.cz, jiri.cerny@ibt.cas.cz; Tel.: +420 325 873 738

check for updates

## 1. Methods : Analysis of the p-PMF data for both protein- and DNA-data

### 1.1. Analysis of the pseudo-potentials of mean force (p-PMF) for proteins from the Protein Data Bank

We analyzed the PDB-data through an averaging procedure as described before to generate the radial distribution functions $g(r)$ and the resulting pseudo potentials of mean force (p-PMF) (see Figure S2), with the use of the approximation of homogeneity in each crystal unit cell and the number of atoms in each PDB-structure. As stated in the main text, we introduce a systematic error into each of the distribution functions as well as into each p-PMF. We mention that the analysis has been performed over the backbone atoms C, N, O and C$\alpha$ over a set of non-redundant X-ray structures with a resolution higher than 2.5 Å. In the analysis of the radial distribution function $g(r)$, we observe bonded contributions along the backbone in the range of r between 0 and 0.4 nm, which are approximately equivalent for all amino-acids within the same residue (see Figure S2 a) ($g(r)$ values ranging from $10^{-6}$ to $10^{-3}$). We observe a larger heterogeneity in the range of r between 0.2-0.8 and 0.3-0.7 nm, corresponding to hydrogen-bonding and sidechain interactions, where sidechain-sidechain interactions (salt-bridges, hydrogen-bonded interactions, van der Waals and aromatic interactions) lead to an effective attraction of the atoms in the backbone. Beyond a distance larger than 1 nm, the effective probability to find another atom becomes continuous, while we correlate relative deviations in the range of $\pm 5 \times 10^{-10}$ as a statistical noise. The effective value of approximately $10^{-9}$ of the

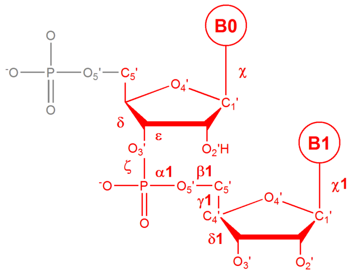


**Fig. S1.** Dihedral angle definitions for the assignment of DNA-configurations to the structural alphabet of DNA. A combination of 9 dihedral angle values as defined in the structural alphabet for DNA is used for the assignment to 44 individual classes [1,2,3]. We applied that set of dihedral angles to the time-dependent assignment of DNA-conformers in the simulations of the Dickerson-Drew DNA-dodecamer as described in the PDB-structure : 1bna.

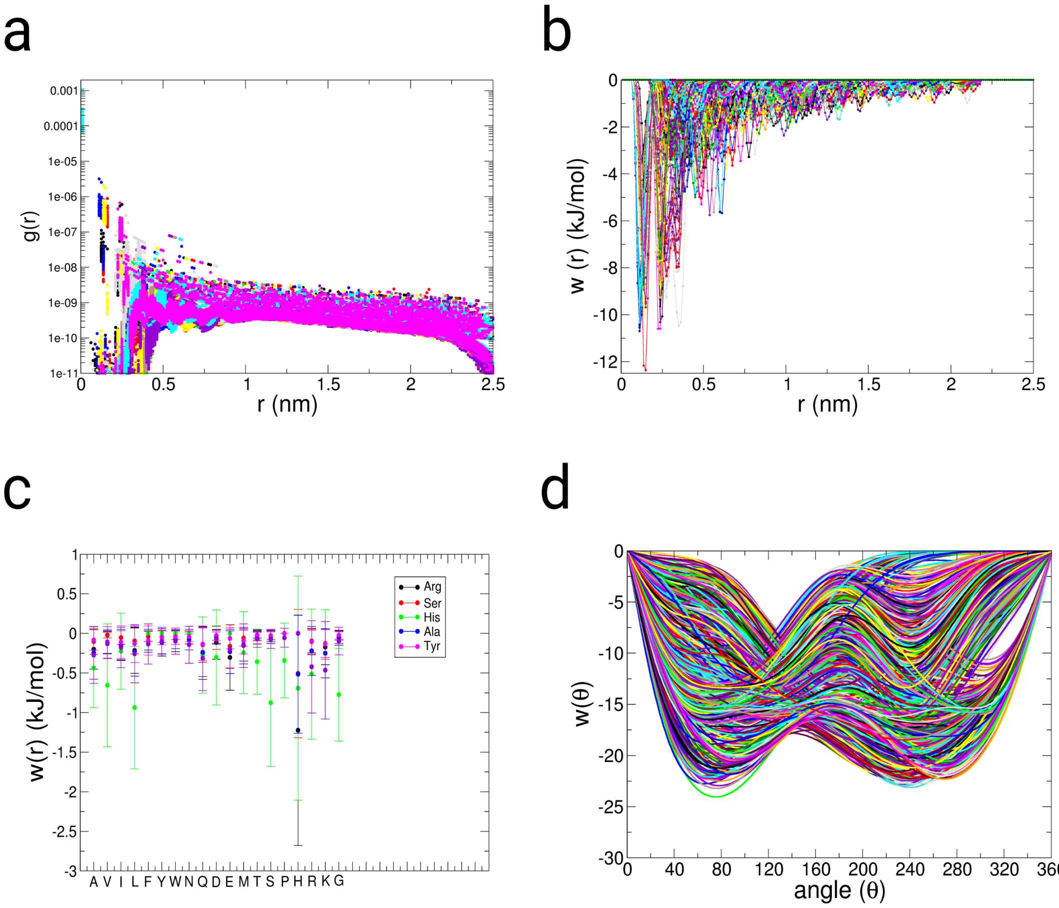


**Fig. S2.** Results of the *pseudo* potential of mean force (p-PMF-$w(r)$) data-analysis of protein data bank (PDB) for peptides and proteins (We selected a set of over 24,300 protein structures, with the criteria that all structures have been determined with a crystallographic resolution higher than 2.5 Å, the sequence length is larger than 50 residues, no DNA- or RNA is present in the system and that the set of structures contains less than 70 % sequence identity.) for 20 individual amino-acids along the backbone-atoms (C, N, C$\alpha$, O) in the form of pair-interactions. The generation of the p-PMFs datasets has been made using the approximation of a uniformity in the PDB-data sets ( a homogeneity in all thermodynamic quantities allowing the determination of a statistical potential of mean force). We take into account that we introduce a systematic error $\delta w(r_{ij})$ into each p-PMF through the approximation. The radial pair-correlation function ($g(r_{ij})$) was measured as function of the pairwise distance $r$ between 2 backbone atoms. The data was deposited as function of the residue and atomic identity, while we used the relation $w(r) = -k_B T \ln(g(r_{ij}))$. The data collection has been performed over a range in the distance $r_{ij}$ from 0 to 2.5 nm. (a) Radial distribution function $g(r_{ij})$ as function of the distance (raw data) averaged over the PDB. (b) p-PMF as function of the distance for all types of 20 different aminoacids over the backbone atoms ($i, j$ = C, N, C$\alpha$, O). (c) Cross-section of the p-PMF within a range in the distance from 0.3 to 0.7 nm for 6 different amino-acid types (Arg, Ser, His, Ala, Tyr, Glu). (d) Torsion p-PMF $w(\theta)$ along the torsion angles $\theta_k = \Phi, \Psi$.

$g(r)$ beyond a distance r of 1 nm indicated a constant $c$ equal to $10^{-9}$ used to normalize the radial distribution function according to the condition expressed in the equation 5 (see Section Methods - Main text). Using the definition for the effective potential of mean force $w(r)$ (p-PMF) for the pair-interaction, we obtained the resulting interaction energies from the $g(r)$-data (see equation 7 in the main text) (see Figure S2 b). The effective interaction energies are quite weak compared to bond-dissociation energies and range from -12 to approximately -8 kJ/mol for the bonded interactions, while the non-covalent interactions are between -8 and -3 kJ/mol within a range in the distance from 0.4 to 1 nm. We relate the region beyond 1 nm to a region which is continuous in energy with a lower structural heterogeneity. We note that secondary structural elements as well as tertiary alignments of non-covalently interacting amino-acid chains occur in the distance-range from 0.4 to 1 nm, which leads to our observation of the largest heterogeneity in that region. We performed an analysis of the cross-section of the average energy value including the standard deviation in the distance-range between 0.4 and 1 nm (see Figure S2 c) as function of the amino-acid index, where we observe that the interactions are amino-acid specific and dependent on the nature of the underlying amino-acid sidechain. The energy-differences defining the heterogeneity lie in between 0.1 and 0.3 kJ/mol except the aminoacid Histidine (including the standard deviation, the energy differences are approximately $\pm$0.5 kJ/mol. In a very broad perspective, that means that protein structure and dynamical properties are controlled by pairwise energy differences of approximately 0.1 to 0.3 kJ/mol along the backbone of proteins.

The radial distribution function $g(r)$ contains a 3-dimensional structural information of the partition of atoms around a central atom including a related p-PMF $w(r)$ to express the effective interaction. Thus, we additionally added the torsional p-PMFs along each of the torsional angles $\Phi$ and $\Psi$ for each aminoacid over the same data-set (see the schematic Figure 1 in the main text and the section Methods). Most of the patterns along the distance dependent distribution are reproducing known amino-acid interaction patterns, while we found surprising differences in the levels of interaction, as for example in the case of Ala in contrast to the rest of aliphatic aminoacids, as well as for hydrophilic aminoacids with a different level of attraction of Asp than Glu for binding to Lys. We mention that already a number of works estimated the interaction energies of aminoacids in proteins using X-ray structural data deposited in the PDB through radial and angular distribution functions [4]. A very recent work highlighted the role of hydrophobic residues (Val, Ile, Leu, Phe, Met, Tyr and Trp) over a wide range of the Uniprot/Swissprot dataset of 549,832 proteins and the related extent of disorder over annotated and non-annotated proteins using a Hydrophobic Cluster Analysis (HCA) [5]. Finally, we also note that similar approaches in coarse-grained descriptions of molecular interactions for MD- and Monte Carlo simulations exist [6,7,8]. A reduced, but an analogous analysis of $g(r)$-data averaged over the PDB has been performed for parts of the aliphatic group of aminoacids [9].

### *1.2. Analysis of pseudo potentials of mean force (p-PMF) for DNA from the Protein Data Bank*

We analyzed the radial distribution functions $g(r)$ and generated the p-PMF-data as function of the pairwise distances between the atoms P, N1/N9, C4/C2, C4′, C5′, O5′ (see Figure S3), while we use the approximation of homogeneity in each crystal unit cell and the number of atoms in each PDB-structure. As stated in the main text, we introduce a systematic error into each of the distribution functions as well as into each p-PMF. In the cumulative plot of all pairwise RDFs, we observe a sinoidal curvature of the pair-correlation functions with alternating maxima at distance-differences of approximately 0.5 nm , which is a characteristic distance between steps (DNA sequence neighbors), while the sinoidal curvature indicates that also a wide range of disordered configurations exists along DNA-sequences (see Figure S3 a). The sinoidal curvature also indicates a high propensity for the DNA to occur in a helical form in which the subsequent steps occur in a stacked order. The sinoidal curvature is also visible in minima in the associated p-PMF ($w(r)$) at the same distance $r$ as in the pair-correlation function $g(r)$ (see Figure S3 b). At the same time, the observation of the shape of the

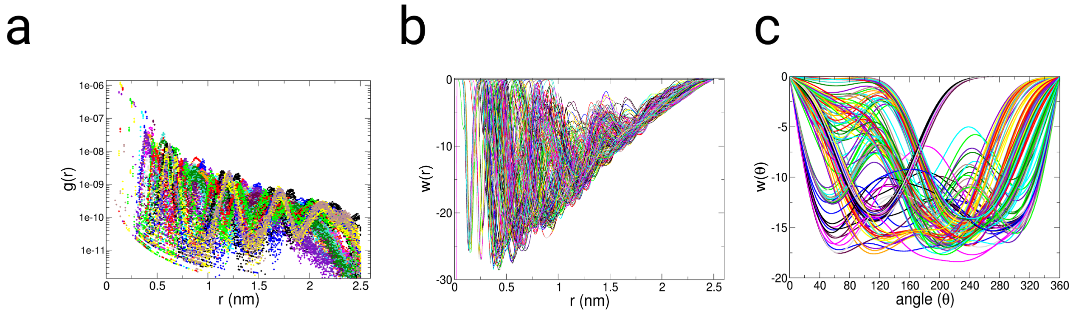


**Fig. S3.** Results from the data-collection over 1,800 X-ray structures of DNA in the PDB. We collected the data using in-house programs through a calculation of inter-atomic distances, we generated radial-distribution functions $g(r)$ and determined the corresponding *pseudo* potentials of mean force $w(r)$ (p-PMF) from that data-set (using the approximation of a uniformity in the PDB data allowing the determination of the potentials of mean force, if the number of atoms, volumes and other thermodynamic quantities are identical between the individual PDB structures). We take into account that we introduce a systematic error $\delta w(r)$ into each p-PMF through the approximation. (a) Radial distribution functions $g(r)$ as function of the pair-wise distances $r_{ij}$ between the atoms P, N9/N1, C4/C2, C4′, C5′, O5′ for the nucleotides containing : Adenine (DA), guanine (DG), cytosine (DC) and thymine (DT). (b) p-PMF $w(r)$ as function of the pair-wise distances $r_{ij}$ between the atoms P, N9/N1, C4/C2, C4′, C5′, O5′ for the nucleotides containing : Adenine (DA), guanine (DG), cytosine (DC) and thymine (DT). (c) Torsion p-PMF $w(\theta)$ along the torsion angles $\theta_k = \alpha, \beta, \gamma, \epsilon, \delta, \chi, \zeta$.

RDF indicates that the DNA-backbone allows in general conformations within a distance range of 0.5 nm , while the configurations in between the sinoidal maxima are related to states higher in energy, which might only be accessed either in rare thermal fluctuations or upon binding to a DNA-binding molecule, such as a transcription factor [10]. The correlations are comparatively long-ranged over a distance of 2.5 nm , leading to attractive interactions even at distances beyond 2.0 nm with energies of approximately -5 kJ/mol (see Figure S3 b).

A large number of structural analyses indicated that DNA can adopt more than the originally suggested canonical B-form [11]. The importance of conformational sub-states in the DNA upon binding of proteins to the minor-groove of DNA has been shown in a variety of structural analyses of protein-DNA complexes [12,13,14]. As indicated in the structural alphabet of DNA, which contains 45 different structural classes, an abundance of configurations exist, which are different from the conventional A-, B- and Z-forms [1,2,3]. The large heterogeneity of states among possible DNA-conformers is also reflected in the variety of structures, which DNA can adopt [15]. Our finding of maxima along the p-PMF partitions with regular repeats of approximately 0.5 nm is in agreement with pre-dominant forms of DNA-configurations, while we observe that sub-populations are associated with higher energies, which can be found in between the regular maxima. Overall, the pair-correlation functions of DNA indicate a long-ranged correlation induced by the general helical form of DNA. Finally, we mention our observation of a general difference in the correlation behavior of purine and pyrimidine bases, which can be divided into 2 general groups regarding their interaction energies of the backbone to the bases.

### *1.3. Penta-alanine configurations in dependency of the position of one Ser residue*

We investigated the influence of a local heterogeneity on a larger pentapeptide, in order to evaluate the effect of the applied PDB-data in the PMF-enriched sampling and the influence of the position of a local mutation to Ser on the conformations of the complete system (see Figure S6 and Figures S7-S13). Penta-alanine has been used as a model system in previous studies, since it mimicks the smallest possible system, which is able to adopt all secondary structure elements [16,17,18,19,20]. We simulated that system using path- and PMF-enriched sampling. The path-sampling result leads to populations of $\alpha$-helical (3) (constant over all 5 residues, $\Delta$F -9 to -10 $k_BT$), partially $\beta$-stranded (1) (residues 2

to 5, ΔF -7 to -9 $k_B T$) and Poly-proline (2) (residues 3 to 5, ΔF -7 to -9 $k_B T$) populations along the FEL as function of Φ and Ψ, which indicates a heterogeneity of Φ and Ψ-conformations already for a homogeneous sequence. Those results are also reflected by a minimum in the $RMSD_{C\alpha-C\alpha}$ to the final structure with $\Delta F = -5\ k_B T$ at a radius of gyration Rg equal to 0.375 nm (see Figure S6). We note that in the path-sampling, we only observe a $L\alpha$-conformation (4, 5) for the C-terminal residue, indicating a barrier for the non-terminal residues to perform that dihedral rotation. The PMF-enriched simulations on the same system show the same tendency for populations at minima corresponding to $\alpha$-helical, $\beta$-stranded, but also populations of $L\alpha$ for residues 2, 3 and 4 with related energies ranging from -5 to -6 $k_B T$. In a subsequent set of simulations, we modeled penta-alanine with Ala-Ser mutations at all 5 different positions from the C- to the N-terminal part. In path-sampling as well as in the PMF-enriched simulations, the populations are shifted in dependency from the position of Ser in the sequence of penta-alanine. In general, the Ala-Ser mutation changes the patterns of the FEL as function of Φ and Ψ towards a population of states with a larger occupancy in the $\beta$- and Poly-Proline helical part with an increasing number of states for the N-terminal mutations Ace-SAAAA-NMe and Ace-ASAAA-NMe (see Figure S16-S20). The change in the populations becomes more pronounced in the systems sampled with the PMF-enriched method, where the largest increase in the heterogeneity is visible for Ace-SAAAA-NMe and Ace-ASAAA-NMe in all regions of the FEL. We analyzed the changes in the minima ((1)-$\beta$-stranded, (2)-PolyProline helical) for the path-sampling simulations between Ace-AAAAA-NMe and the 5 mutations and find an increase in the populations of minima (1) and (2) for all mutations, with the largest energy difference to pentaalanine in the case of the N-terminal Ala-Ser mutation Ace-SAAAA-NMe. The effects of the mutations on the populations of the minima at (3), (4) and (5) are a shift of the population density from ((3) $\alpha$-helical) towards $\beta$-stranded and PolyProline configurations, while minima (4, 5) are not populated. The relative shifts in the populations are expressed in a shift in the energy by 1-2 $k_B T$. In the PMF-sampling simulations, we observe the same shifts as in the path-sampling simulation with a higher population of states (1, 2) away from $\alpha$-helical (3) configurations, while also states $L\alpha$ (4, 5) are populated with an increasing propensity. That means that the PMF-enriched sampling changes the effective energy landscape according to a system-dependent potential of mean force $w(r)$, which lowers the effective barrier for the system to change its states from (1, 2, 3) to (4, 5). The relative shifts between (1, 2) and (3) are associated with energy changes by 1-2 $k_B T$, while changes at $L\alpha$ are associated with larger energies in the range of up to 5 $k_B T$. In the assignment of conformers to the structural alphabet of proteins, we observe the population of $\alpha$-helical conformations (conformer k (N-cap $\alpha$)) in the path-sampling simulations over the simulation time of 10 ns, while conformers a and b occur with a minor occurrence for the pentapeptides AAAAA, AASAA and SAAAA (see Figure S6). In contrast, we observe in the PMF-enriched simulations that the populations are shifted in depdendency of the Ser position in the sequence, with an increasing probability for extended conformations (conformer l (C-cap $\alpha$)) in the case of SAAAA (see Figures S12 and S6).

We conclude that we find an increasing shift in the population densities depending from the position of Ser in comparison with penta-alanine, while we observe that the largest changes are associated with a N-terminal Ala-Ser mutation in Ace-SAAAA-NMe. While penta-alanine populates primarily $\alpha$-helical conformers, the $\alpha$-helical stability decreases and relocates towards populations corresponding to $\beta$- and PolyProline-helical conformers. We attribute this effect to the averaging over a broad PDB-population containing multiple possible secondary structure elements. In contrast to the shifts in the population we have observed for the pentapeptides in combination with Ser, a mutation with Trp leads to a stabilized $\alpha$-helix due to its sidechain interaction [17]. Experimentally, it has been found that the alanine-rich octapeptide AGAAAAGA belongs to a highly potent amyloidogenic species of the syrian hamster prion-protein ShPrP (113-120), where a strong $\beta$-strand propensity is induced by the 2-Gly residues at the positions 2 and 7 in the sequence [19]. Our observation of a perturbation of the overall structure away from the $\alpha$-helical conformation, especially for the N-terminal position of Ser in the sequence of the peptide SAAAA is in agreement with that finding. We

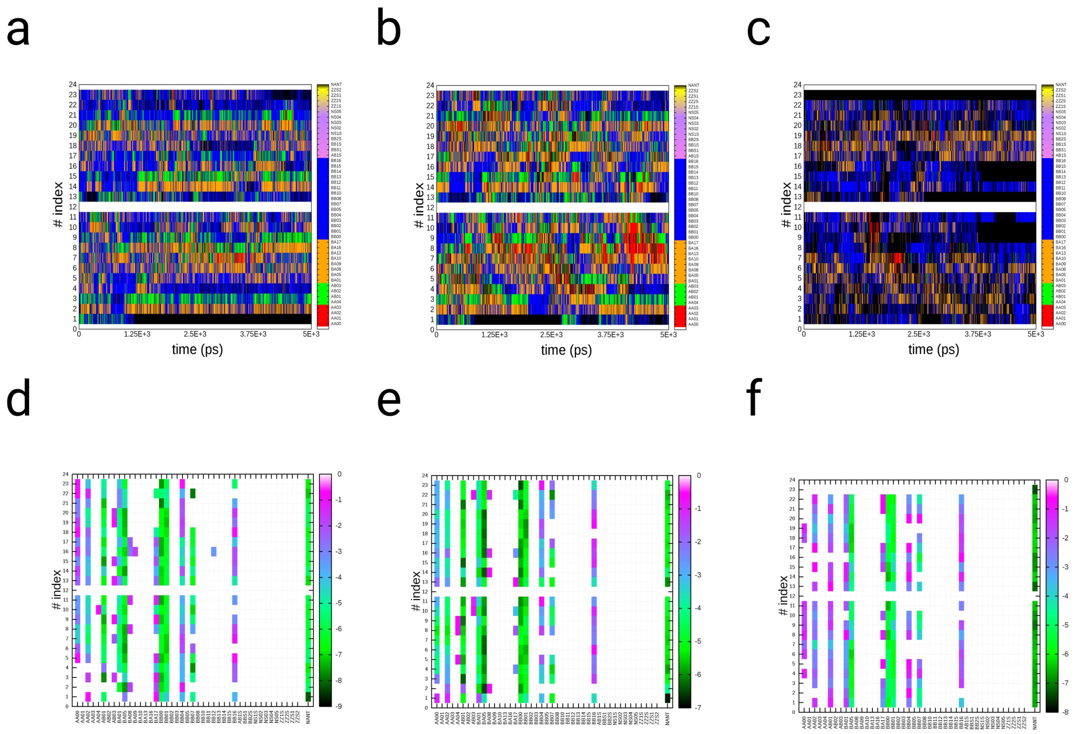


**Fig. S4.** Results from the assignment-analysis of the 3 trajectories of the Dickerson-Drew DNA dodecamer using the pseudo potentials of mean force directly without the definition of the corresponding partitions $\Omega_i$. Here we applied a coupling strength $\alpha$ of $10^{-4}$. In the assignment analysis a combination of 9 dihedral angle values as defined in the structural alphabet for DNA is used for the assignment to 44 individual classes [1,2,3] (Each single of the 44 classes is expressed by 4 letters, the class 45 corresponds to the non-assigned class (NANT), where the combination of 9 torsions cannot be assigned to any of the 44 classes). (a, b, c) Structural classes as function of time from the simulation using different conventional forcefields $(H(A))$ : AMBER99 (a), AMBER12sb (b) and AMBER14sb (c). (d, e, f) Free energy partition ($\Delta F = -k_B T \ln(p/p_{min})$) as function of the conformer class index and the step-index along the DNA-sequence from the simulations using AMBER99 (d), AMBER12sb (e) and AMBER14sb (f). The color bar expresses the energies in units of $k_B T$. (For the studied dodecamer residues 1 to 12 form the first DNA strand (chain A in the 1bna) and residues 13 to 24 belong to the second strand (chain B). Within each strand containing 12 nucleotide residues there can be assigned 11 dinucleotide steps numbered from 1 to 11 for the first strand and 13 to 23 for the second. The step-index #N then corresponds to a dinucleotide formed by residues N and N+1.).

also find qualitative agreement with a Raman spectroscopic study on the influence of point mutations within an $(AG)_n$-sequence with Tyr, Val or Ser showed the effect of the position and the chemical nature of the mutation on the conformations of the systems, while especially N-terminal modifications had the largest inflence on the stability of the conformer [21].

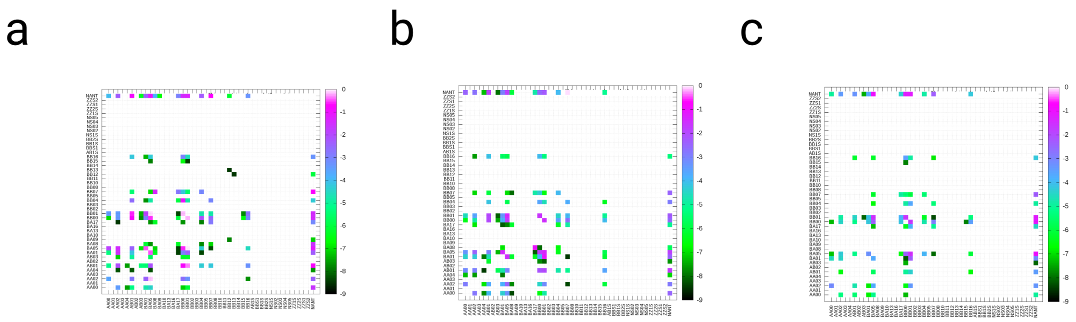

**Fig. S5.** Analysis of the 3 trajectories of the Dickerson-Drew DNA dodecamer using the *pseudo* potentials of mean force p-PMF directly without the definition of the corresponding partitions $\Omega_i$. Here we applied a coupling strength $\alpha$ of $10^{-4}$. Kinetic analysis of the frequencies $\ln \nu$, $\nu = 1/\tau$ of the transition time between different structural classes (in the order from a class n shown on the x-axis to classes n′ on the y-axis) as function of the class index (1-44 (class #45 represents the non-assigned class)). Panel (a) shows the result from the simulation with AMBER99, (b) using AMBER12sb and (c) with AMBER14sb.

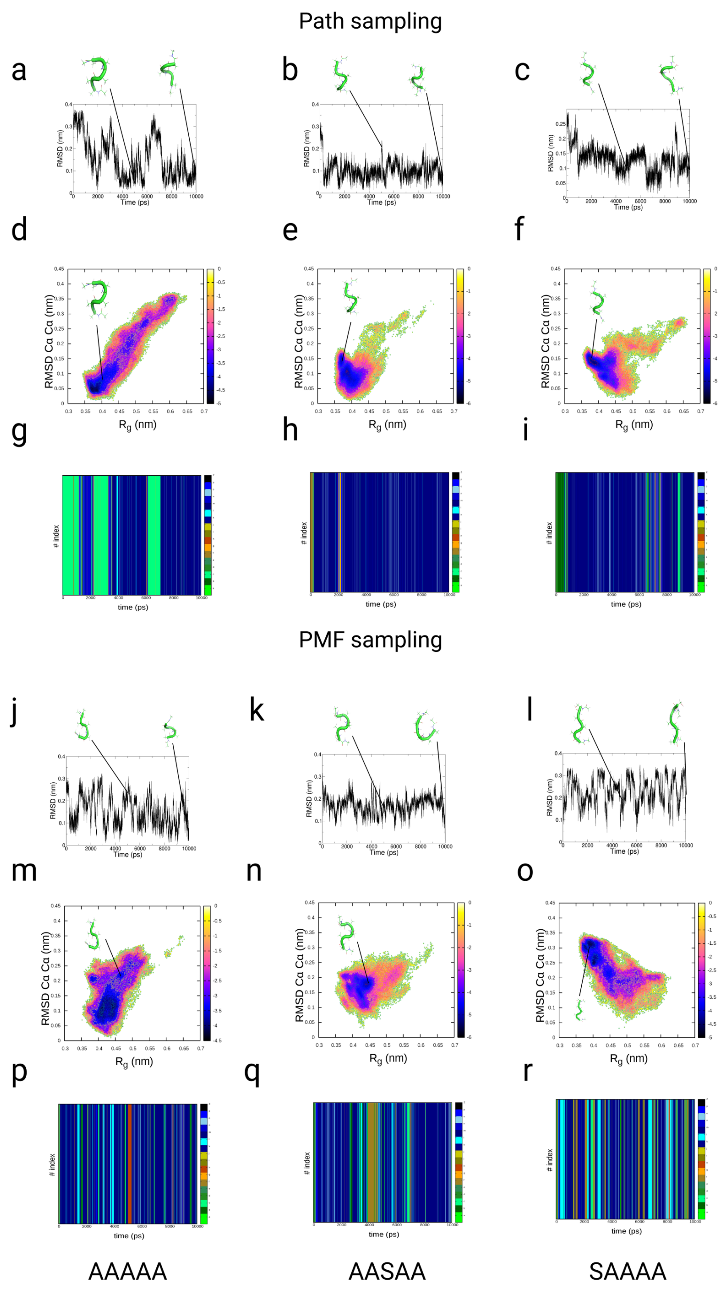


**Fig. S6.** Results from path-sampling and PMF-sampling simulations of the pentapeptides : (a, d, g, j, m, p) AAAAA, AAAAS, (b, e, h, k, n, q) AAASA, AASAA, ASAAA and (c, f, i, l, o, r) SAAAA (AAAAA, AASAA and SAAAA shown in this figure. See the supplementary Figures 15S-21S for the data related to the other peptides). (a, b, c) $RMSD_{C\alpha-C\alpha}$ to the final structure as function of simulation time (path-sampling) (a: AAAAA, b: AASAA, c: SAAAA). (d, e, f) Projection of free energy on the $RMSD_{C\alpha-C\alpha}$ and radius of gyration Rg (path-sampling). (g, h, i) Results from assignment of conformers in each MD-trajectory to the structural alphabet for proteins [22]. (j, k, l) $RMSD_{C\alpha-C\alpha}$ to the final structure as function of simulation time (PMF-sampling). (m, n, o) Projection of free energy on the $RMSD_{C\alpha-C\alpha}$ and radius of gyration Rg (PMF-sampling). (p, q, r) Results from assignment of conformers in each PMF-trajectory to the structural alphabet for proteins [22]. We observe a shift in the distribution of the minima along the FEL in dependency of the position of the Ser-residue in the sequence of the pentapeptide in simulations with both methods. In the PMF-based sampling, the structural heterogeneity is larger due to the anisotropy of the applied PMF, which is constructed from the PDB-data for each individual pentapeptide. That procedure leads to a heterogeneous landscape in dependency of the position of the aminoacid in the sequence, and leads to stronger shifts in the structural changes of the pentapeptide in dependency of the position of the Ser-mutation.

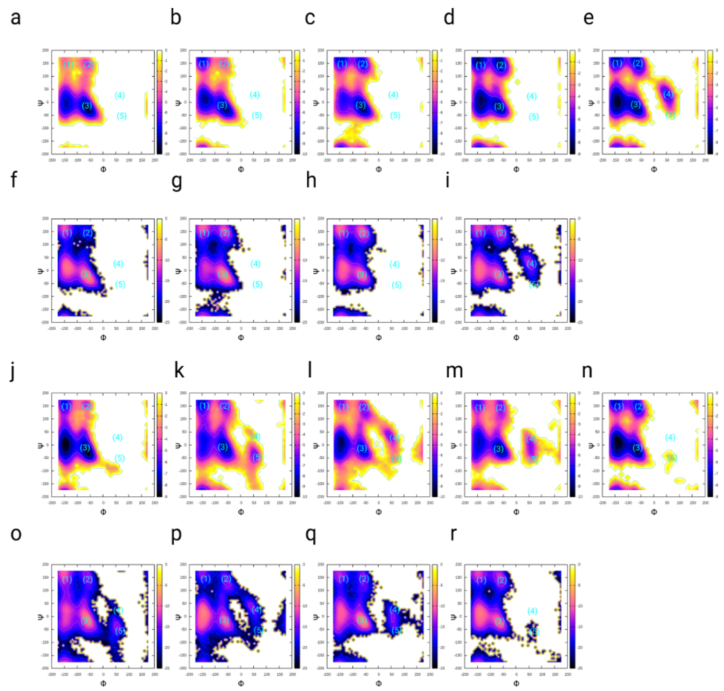


**Fig. S7.** Results from path- and PMF-sampling simulations of Penta-alanine Ace-Ala-Ala-Ala-Ala-Ala-NMe using the AMBER99 forcefield. (a-e) Free energy landscapes projected on the backbone dihedrals Φ and Ψ along the index of the polypeptide from the path sampling simulation (a=N-terminal position, to e=C-terminus). (f-i) Correlated probabilities $P(A, B) = P(\Phi_1, \Psi_1)P(\Phi_2, \Psi_2)$ of the probabilities $P(\Phi_1, \Psi_1)$ and $P(\Phi_2, \Psi_2)$ along the index of the polypeptide between neighboring FELs shown in panels (a-e). (j-n) Free energy landscapes projected on the backbone dihedrals Φ and Ψ along the index of the polypeptide from the PMF-sampling simulation (j=N-terminal position, to n=C-terminus). (o-r) Correlated probabilities $P(A, B) = P(\Phi_1, \Psi_1)P(\Phi_2, \Psi_2)$ of the probabilities $P(\Phi_1, \Psi_1)$ and $P(\Phi_2, \Psi_2)$ along the index of the polypeptide between neighboring FELs shown in panels (j-n).

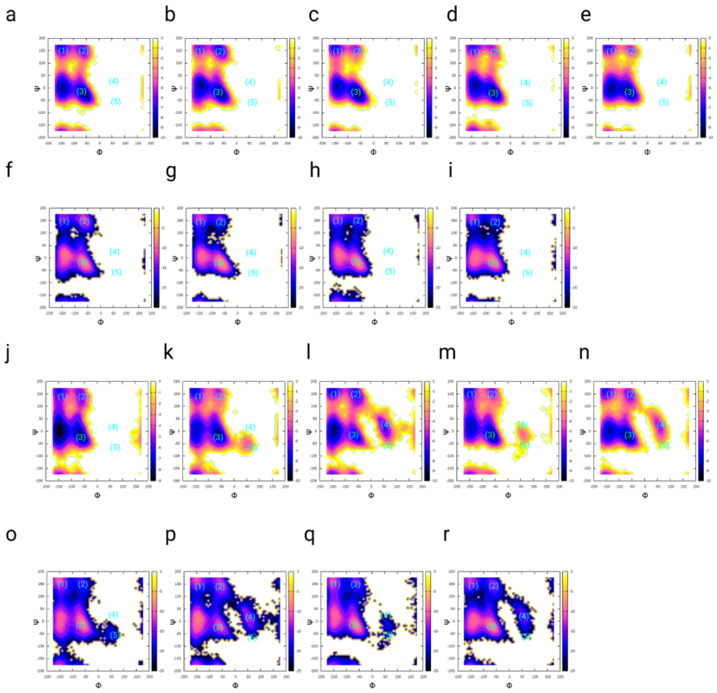


**Fig. S8.** Results from path- and PMF-sampling simulations of Penta-alanine with one Ala-Ser mutation : Ace-Ala-Ala-Ala-Ala-Ser-NMe using the AMBER99 forcefield. (a-e) Free energy landscapes projected on the backbone dihedrals $\Phi$ and $\Psi$ along the index of the polypeptide from the path sampling simulation (a=N-terminal position, to e=C-terminus). (f-i) Correlated probabilities $P(A, B) = P(\Phi_1, \Psi_1)P(\Phi_2, \Psi_2)$ of the probabilities $P(\Phi_1, \Psi_1)$ and $P(\Phi_2, \Psi_2)$ along the index of the polypeptide between neighboring FELs shown in panels (a-e). (j-n) Free energy landscapes projected on the backbone dihedrals $\Phi$ and $\Psi$ along the index of the polypeptide from the PMF-sampling simulation (j=N-terminal position, to n=C-terminus). (o-r) Correlated probabilities $P(A, B) = P(\Phi_1, \Psi_1)P(\Phi_2, \Psi_2)$ of the probabilities $P(\Phi_1, \Psi_1)$ and $P(\Phi_2, \Psi_2)$ along the index of the polypeptide between neighboring FELs shown in panels (j-n).

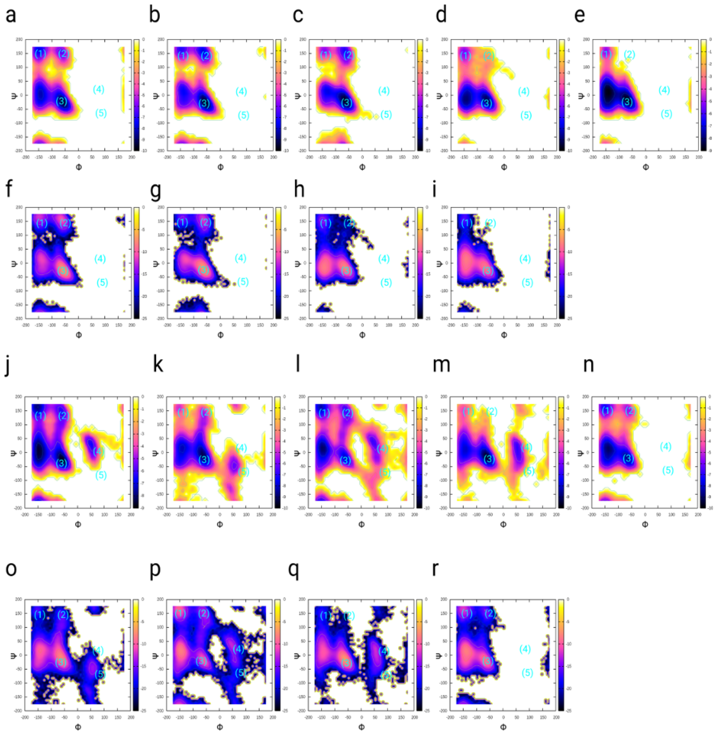

**Fig. S9.** Results from path- and PMF-sampling simulations of Penta-alanine with one Ala-Ser mutation : Ace-Ala-Ala-Ala-Ser-Ala-NMe using the AMBER99 forcefield. (a-e) Free energy landscapes projected on the backbone dihedrals $\Phi$ and $\Psi$ along the index of the polypeptide from the path sampling simulation (a=N-terminal position, to e=C-terminus). (f-i) Correlated probabilities $P(A, B) = P(\Phi_1, \Psi_1)P(\Phi_2, \Psi_2)$ of the probabilities $P(\Phi_1, \Psi_1)$ and $P(\Phi_2, \Psi_2)$ along the index of the polypeptide between neighboring FELs shown in panels (a-e). (j-n) Free energy landscapes projected on the backbone dihedrals $\Phi$ and $\Psi$ along the index of the polypeptide from the PMF-sampling simulation (j=N-terminal position, to n=C-terminus). (o-r) Correlated probabilities $P(A, B) = P(\Phi_1, \Psi_1)P(\Phi_2, \Psi_2)$ of the probabilities $P(\Phi_1, \Psi_1)$ and $P(\Phi_2, \Psi_2)$ along the index of the polypeptide between neighboring FELs shown in panels (j-n).

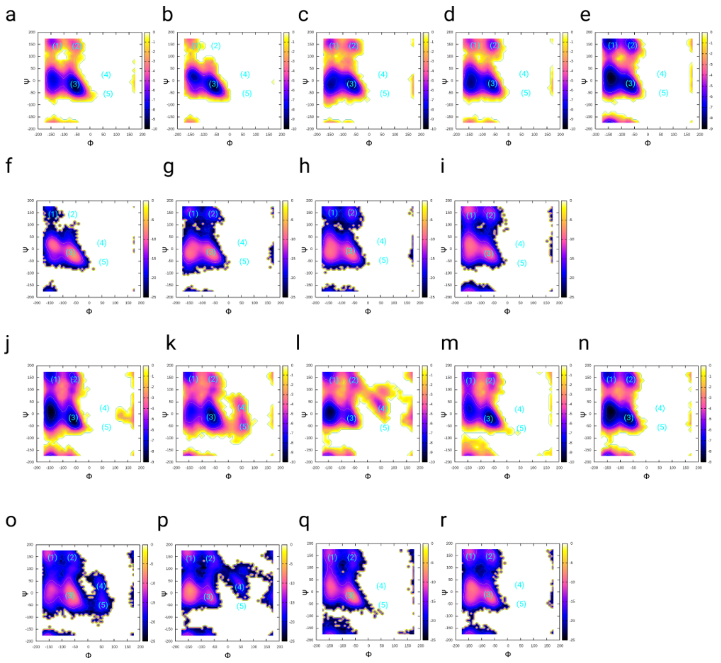


**Fig. S10.** Results from path- and PMF-sampling simulations of Penta-alanine with one Ala-Ser mutation : Ace-Ala-Ala-Ser-Ala-Ala-NMe using the AMBER99 forcefield. (a-e) Free energy landscapes projected on the backbone dihedrals $\Phi$ and $\Psi$ along the index of the polypeptide from the path sampling simulation (a=N-terminal position, to e=C-terminus). (f-i) Correlated probabilities $P(A, B) = P(\Phi_1, \Psi_1)P(\Phi_2, \Psi_2)$ of the probabilities $P(\Phi_1, \Psi_1)$ and $P(\Phi_2, \Psi_2)$ along the index of the polypeptide between neighboring FELs shown in panels (a-e). (j-n) Free energy landscapes projected on the backbone dihedrals $\Phi$ and $\Psi$ along the index of the polypeptide from the PMF-sampling simulation (j=N-terminal position, to n=C-terminus). (o-r) Correlated probabilities $P(A, B) = P(\Phi_1, \Psi_1)P(\Phi_2, \Psi_2)$ of the probabilities $P(\Phi_1, \Psi_1)$ and $P(\Phi_2, \Psi_2)$ along the index of the polypeptide between neighboring FELs shown in panels (j-n).

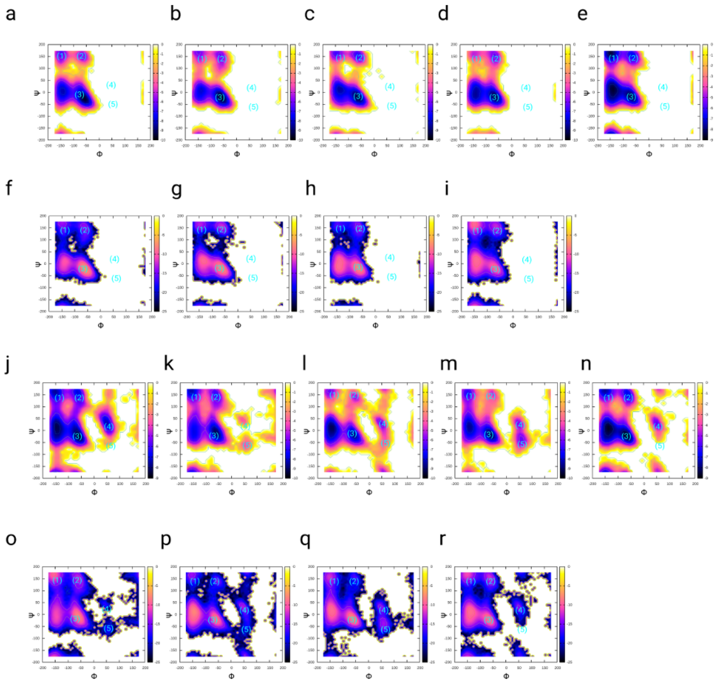


**Fig. S11.** Results from path- and PMF-sampling simulations of Penta-alanine with one Ala-Ser mutation : Ace-Ala-Ser-Ala-Ala-Ala-NMe using the AMBER99 forcefield. (a-e) Free energy landscapes projected on the backbone dihedrals $\Phi$ and $\Psi$ along the index of the polypeptide from the path sampling simulation (a=N-terminal position, to e=C-terminus). (f-i) Correlated probabilities $P(A,B) = P(\Phi_1,\Psi_1)P(\Phi_2,\Psi_2)$ of the probabilities $P(\Phi_1,\Psi_1)$ and $P(\Phi_2,\Psi_2)$ along the index of the polypeptide between neighboring FELs shown in panels (a-e). (j-n) Free energy landscapes projected on the backbone dihedrals $\Phi$ and $\Psi$ along the index of the polypeptide from the PMF-sampling simulation (j=N-terminal position, to n=C-terminus). (o-r) Correlated probabilities $P(A,B) = P(\Phi_1,\Psi_1)P(\Phi_2,\Psi_2)$ of the probabilities $P(\Phi_1,\Psi_1)$ and $P(\Phi_2,\Psi_2)$ along the index of the polypeptide between neighboring FELs shown in panels (j-n).

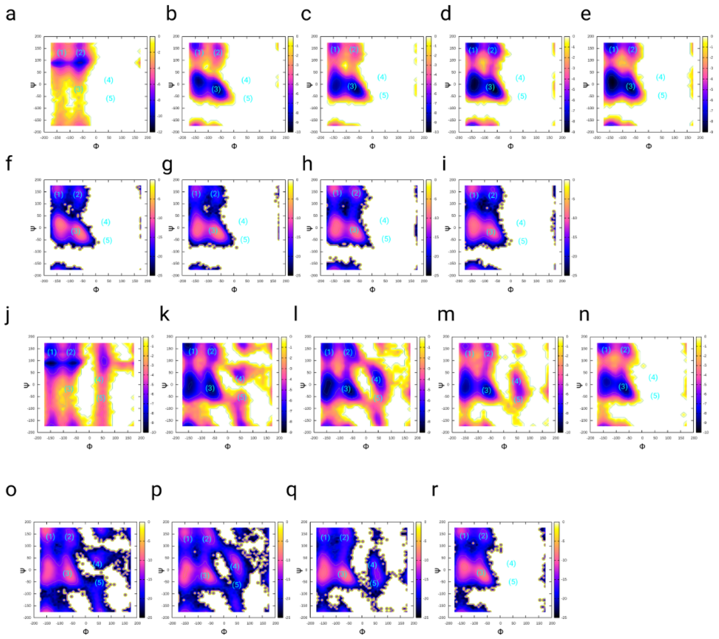


**Fig. S12.** Results from path- and PMF-sampling simulations of Penta-alanine with one Ala-Ser mutation : Ace-Ser-Ala-Ala-Ala-Ala-NMe using the AMBER99 forcefield. (a-e) Free energy landscapes projected on the backbone dihedrals $\Phi$ and $\Psi$ along the index of the polypeptide from the path sampling simulation (a=N-terminal position, to e=C-terminus). (f-i) Correlated probabilities $P(A, B) = P(\Phi_1, \Psi_1)P(\Phi_2, \Psi_2)$ of the probabilities $P(\Phi_1, \Psi_1)$ and $P(\Phi_2, \Psi_2)$ along the index of the polypeptide between neighboring FELs shown in panels (a-e). (j-n) Free energy landscapes projected on the backbone dihedrals $\Phi$ and $\Psi$ along the index of the polypeptide from the PMF-sampling simulation (j=N-terminal position, to n=C-terminus). (o-r) Correlated probabilities $P(A, B) = P(\Phi_1, \Psi_1)P(\Phi_2, \Psi_2)$ of the probabilities $P(\Phi_1, \Psi_1)$ and $P(\Phi_2, \Psi_2)$ along the index of the polypeptide between neighboring FELs shown in panels (j-n).

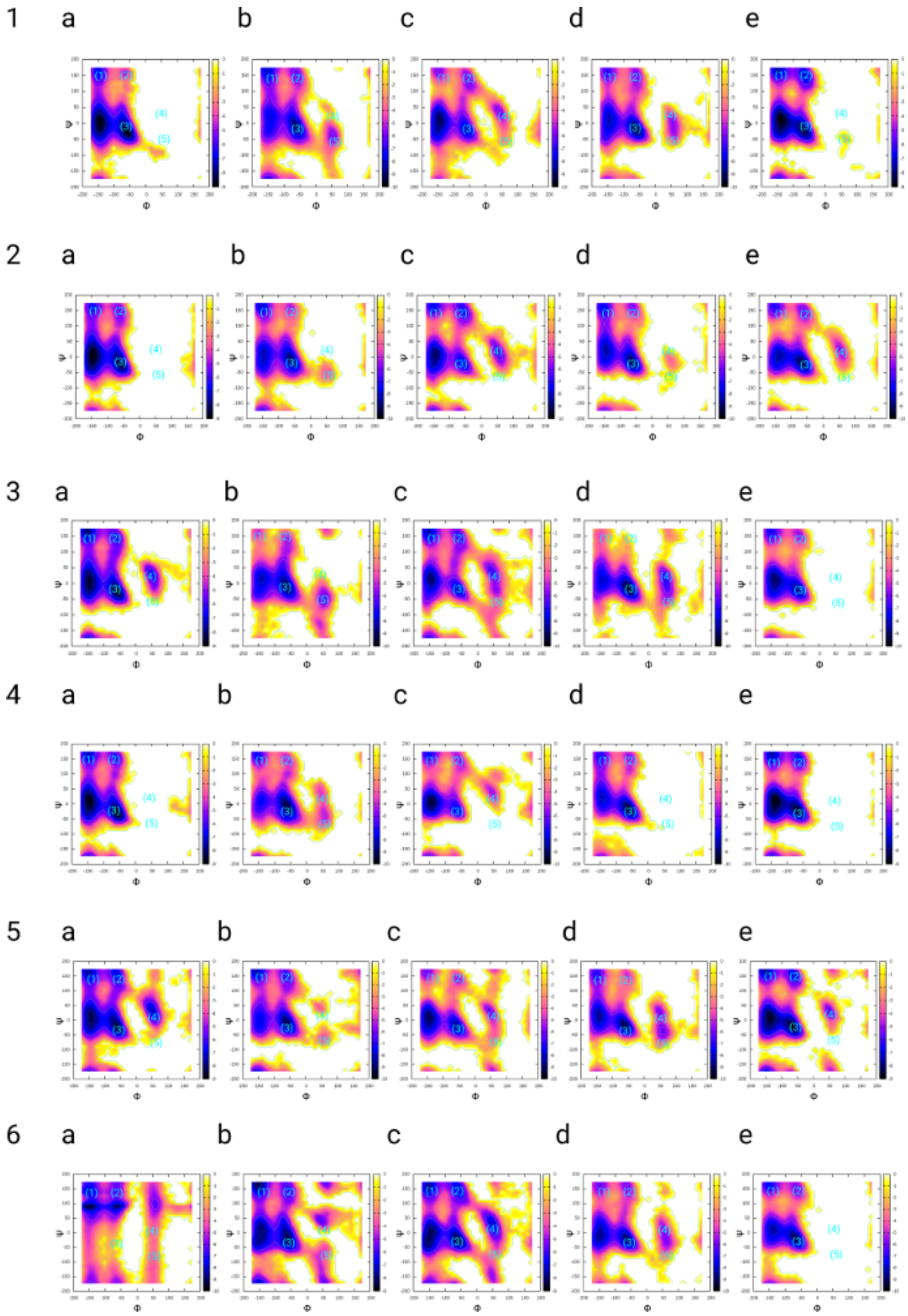

**Fig. S13.** Summary of PMF-sampling simulations for penta-alanine ((1) AAAAA) and the mutated versions : (2) AAAAS, (3) AAASA, (4) AASAA, (5) ASAAA and (6) SAAAA. Free energy landscapes projected on the backbone dihedrals Φ and Ψ along the index of the polypeptide from the path sampling simulation (a=N-terminal position, to e=C-terminus).

[1] Svozil, D.; Kalina, J.; Omelka, M.; Schneider, B. *Nucleic Acids Res.* **2008**, *36*, 3690–3706.
[2] Schneider, B.; Božíková, P.; Nečasová, I.; Čech, P.; Svozil, D.; Černý, J. *Acta Cryst.* **2018**, *D74*, 52–64.
[3] Schneider, B.; Božíková, P.; Čech, P.; Svozil, D.; Černý, J. *Genes (Basel)* **2017**, *8*, 278.
[4] Rackovsky, S.; Scheraga, H. *Proc. Natl. Acad. Sci. U.S.A.* **1977**, *74*, 5248–5251.
[5] Bitard-Feildel, T.; Callebaut, I. *Sci. Rep.* **2017**, *7*, 41425.
[6] Mullinax, J.W.; Noid, W.G. *Proc. Natl. Acad. Sci. U.S.A.* **2010**, *107*, 19867–19872.
[7] Alm, E.; Baker, D. *Proc. Natl. Acad. Sci. U.S.A.* **1999**, *96*, 11305–11310.
[8] Liwo, A.; Olziej, S.; Pincus, M.R.; Wawak, R.J.; Rackowsky, S.; Scheraga, H.A. *J. Comput. Chem.* **1997**, *18*, 849–873.
[9] Kržišnik, K.; Urbic, T. *Acta Chim. Slov.* **2015**, *62*, 574–581.
[10] Morimura, H.; Tanaka, S.; Ishitobi, H.; Mikami, T.; Kamachi, Y.; Kondoh, H.; Inouye, Y. *ACS Nano* **2013**, *7*, 10733–10740.
[11] Watson, J.D.; Crick, F.H.C. *Nature* **1953**, *171*, 737–738.
[12] Tolstorukov, M.Y.; Jernigan, R.L.; Zhurkin, V.B. *J. Mol. Biol.* **2004**, *337*, 65–76.
[13] Moravek, Z.; Neidle, S.; Schneider, B. *Nucleic Acids Res.* **2002**, *30*, 1182–1191.
[14] Oguey, C.; Foloppe, N.; Hartmann, B. *PLoS One* **2010**, *5*, e15931.
[15] Orbons, L.P.; van der Marel, G.A.; van Boom, J.H.; Altona, C. *Nucleic Acids Res.* **1986**, *14*, 4187–4196.
[16] Feng, Y.; Huang, J.; Kim, S.; Shim, J.H.; MacKerell, A.D.; Ge, N.H. *J. Phys. Chem. B* **2016**, *120*, 5325–5339.
[17] Jas, G.S.; Kuczera, K. *Mol. Sim.* **2012**, *38*, 682–694.
[18] Ahn, S.H.; Grate, J.W.; Darve, E.F. *J. Chem. Phys.* **2017**, *147*, 074115.
[19] Ma, B.; Nussinov, R. *Prot. Sci.* **2002**, *11*, 2335–2350.
[20] Debiec, K.T.; Cerutti, D.S.; Baker, L.R.; Gronenborn, A.M.; Case, D.A.; Chong, L.T. *J. Chem. Theor. Comput.* **2016**, *12*, 3926–3947.
[21] Taddei, P.; Asakura, T.; Yao, J.; Monti, P. *Biopolymers* **2004**, *75*, 314–324.
[22] deBevern, A.G.; Etchebest, C.; Hazout, S. *Proteins* **2000**, *41*, 271–287.


© 2018 by the authors. Licensee MDPI, Basel, Switzerland. This article is an open access article distributed under the terms and conditions of the Creative Commons Attribution (CC BY) license (http://creativecommons.org/licenses/by/4.0/).
